# Comprehensive Evaluation of the Efficacy and Safety of the *Clostridioides difficile* Toxoid Vaccine: A Meta‐Analysis

**DOI:** 10.1155/cjid/1160340

**Published:** 2026-07-30

**Authors:** Muhammad Shahzil, Zainab Jamil, Saleha Azeem, Minahel Shehzadi, Aisha Shabbir, Muhammad Saad Faisal, Fariha Hasan, Muhammad Ali Khaqan, Ammad Javaid Chaudhary, Syed-Mohammed Jafri

**Affiliations:** ^1^ Department of Internal Medicine, Milton S. Hershey Medical Center, The Pennsylvania State University, Hershey 17033, Pennsylvania, USA, psu.edu; ^2^ Department of Medicine, King Edward Medical University, Lahore 54000, Punjab, Pakistan, kemu.edu.pk; ^3^ Department of Internal Medicine, University of Minnesota, Minneapolis, Minnesota, USA, umn.edu; ^4^ Department of Internal Medicine, Henry Ford Hospital, Detroit 48202, Michigan, USA, henryford.com; ^5^ Department of Internal Medicine, Cooper University Hospital, Camden 08103, New Jersey, USA, cooperhealth.org; ^6^ Department of Gastroenterology and Hepatology, University of Kentucky, Lexington 40506, Kentucky, USA, uky.edu; ^7^ Department of Gastroenterology and Hepatology, Henry Ford Hospital, Detroit 48202, Michigan, USA, henryford.com

**Keywords:** antitoxin antibodies, humoral immunity, seroconversion, toxoid immunization, vaccine safety

## Abstract

**Background:**

*Clostridioides difficile* infection (CDI) significantly contributes to healthcare‐associated morbidity and mortality. Vaccination against *C. difficile* toxins may provide an effective preventive strategy, yet current data remain inconclusive. This meta‐analysis evaluates the efficacy, immunogenicity, and safety of the *C. difficile* toxoid vaccine.

**Methods:**

We systematically searched PubMed, Embase, Web of Science, and the Cochrane Central Register of Controlled Trials from inception through August 2024. Eligible studies were randomized controlled trials enrolling adults aged 50–85 years that compared genetically detoxified *Clostridioides difficile* toxoid vaccines (50–200 µg) with placebo and reported clinical efficacy, immunogenicity, or safety outcomes. Random‐effects meta‐analyses were performed, with risk of bias assessed using RoB 2.0 and certainty of evidence graded using GRADE. The study was prospectively registered in PROSPERO (CRD42024597465). No external funding was received for this study.

**Results:**

Eight trials (11,717 participants) showed no significant vaccine effect on overall CDI incidence (RR = 0.86; 95% CI 0.56–1.32; I^2^ = 0%) or severe CDI (RR = 0.25; 95% CI 0.02–3.75; I^2^ = 71%), though severe CDI risk trended lower in vaccinated groups. Immunogenicity analyses demonstrated significant seroconversion rates for toxin A (RR = 23.28; 95% CI 7.62–71.16; I^2^ = 0%) and toxin B (RR = 19.23; 95% CI 4.84–76.38; I^2^ = 0%). Vaccine recipients exhibited significantly elevated geometric mean concentrations (GMCs) for antitoxin A and B antibodies across various dosing regimens, particularly at higher doses (200 µg). Local adverse events, including pain (RR = 3.02; 95% CI 2.47–3.70), swelling (RR = 8.53; 95% CI 3.15–23.14), and erythema (RR = 6.78; 95% CI 2.35–19.61), were significantly increased postvaccination, but serious systemic reactions, infections, gastrointestinal disturbances, and mortality rates showed no significant differences compared to placebo. Interpretation of vaccine efficacy is limited by imprecision of effect estimates, heterogeneity in immunogenicity reporting, and the small number of trials evaluating severe CDI outcomes.

**Conclusion:**

*Clostridioides difficile* toxoid vaccines induce strong humoral immune responses and demonstrate an acceptable safety profile but do not significantly reduce the overall CDI incidence. Evidence suggests a potential role in mitigating severe disease; however, clinical benefit remains uncertain due to imprecision and heterogeneity.

## 1. Introduction

*Clostridioides difficile* (*C. difficile*) is a Gram‐positive, endospore‐forming, opportunistic bacterial pathogen that can colonize the human gut under normal conditions; however, disruption of the intestinal microenvironment may allow overgrowth and progression to pathogenic infection. *C. difficile* is a leading cause of infectious diarrhea and pseudomembranous colitis and represents a significant source of morbidity and mortality, particularly in healthcare settings [1]. Older hospitalized adults, especially those aged over 65 years, are at the greatest risk of developing *C. difficile* infection (CDI) due to comorbid medical conditions, age‐related immune dysfunction, and frequent exposure to antibiotics. Alterations of the gut microbiota caused by antibiotic therapy, chemotherapy, and proton pump inhibitors further promote *C. difficile* proliferation and toxin‐mediated disease [1, 2]. Although CDI is most commonly associated with hospitals and long‐term care facilities, increasing rates of community‐acquired infection have also been reported [3].

Pathogenicity of *C. difficile* is restricted to toxigenic strains that produce exotoxins TcdA (toxin A) and/or TcdB (toxin B), which are essential mediators of disease in humans [4]. These toxins inactivate Rho family GTPases, leading to actin cytoskeleton disruption, epithelial injury, inflammatory signaling, and cell death, ultimately resulting in diarrhea and pseudomembranous colitis [5]. Certain strains additionally produce a binary toxin that enhances virulence through irreversible adenosine diphosphate–ribosylation of actin [6]. The clinical spectrum of CDI ranges from asymptomatic carriage and mild‐to‐moderate diarrhea to severe colitis, toxic megacolon, and life‐threatening systemic illness. Severe CDI is often characterized by fever, leukocytosis, hypoalbuminemia, hypovolemia due to gastrointestinal fluid losses, and, in advanced cases, lactic acidosis [7].

Current management of primary CDI relies primarily on antibiotic therapy, including metronidazole, vancomycin, and fidaxomicin [8]. Recurrent disease remains a major challenge and may require prolonged or repeated antibiotic courses, monoclonal antibody therapy such as bezlotoxumab targeting toxin B, or fecal microbiota transplantation from healthy donors [9]. Importantly, most preventive and adjunctive therapeutic strategies focus on neutralizing the effects of toxins A and B rather than preventing bacterial colonization itself [10].

Accumulating evidence supports a central role for humoral immunity in modulating CDI severity and recurrence. Prior studies have demonstrated an inverse association between serum antitoxin antibody levels and the risk of recurrent CDI, suggesting that enhancement of toxin‐specific immune responses may confer clinical benefit [11]. This observation has provided the biological rationale for the development of toxoid vaccines designed to induce neutralizing antibodies against *C. difficile* toxins. Toxoid vaccine strategies aim to spare the gastrointestinal tract while priming the immune system to recognize and neutralize toxins, thereby preventing or attenuating disease manifestations [12]. However, despite their ability to induce antibody‐mediated toxin neutralization, toxoid vaccines may have inherent limitations, as they do not prevent bacterial colonization, cytotoxicity, or spore germination and may theoretically increase asymptomatic carriage [13].

Given that *C. difficile* toxoid vaccines have not yet been approved for clinical use and that available randomized trials have yielded heterogeneous and sometimes inconclusive efficacy results, a comprehensive synthesis of existing evidence is warranted. Therefore, we conducted a systematic review and meta‐analysis of randomized controlled trials to evaluate the safety, immunogenicity, and efficacy of *Clostridioides difficile* toxoid vaccines. This analysis aims to clarify the potential role of toxoid vaccination within the prevention and management landscape of CDI and to inform future research and clinical development strategies.

## 2. Methods

This systematic review and meta‐analysis was conducted in accordance with the Cochrane Handbook for Systematic Reviews of Interventions and is reported following the Preferred Reporting Items for Systematic Reviews and Meta‐Analyses (PRISMA) guidelines [14]. The study protocol was prospectively registered in PROSPERO (CRD42024597465). As this analysis synthesized data from previously published studies, institutional review board approval and informed consent were not required. A comprehensive literature search was performed in the Cochrane Central Register of Controlled Trials (CENTRAL), PubMed, Embase (Elsevier), and Web of Science from database inception through August 2024. The search was restricted to randomized controlled trials. Medical Subject Headings (MeSH) and free‐text terms related to *Clostridium difficile*, *C. difficile*, and vaccination were used. The complete electronic search strategy, including database‐specific search strings, is provided in Supporting File 1.

Eligible studies were randomized controlled trials comparing *Clostridioides difficile* toxoid vaccines with placebo. Inclusion criteria required adult participants aged 50–85 years and evaluation of vaccines composed of detoxified toxins A and B, administered with aluminum hydroxide adjuvant. The comparator was normal saline placebo. Studies were required to report at least one outcome related to vaccine efficacy, immunogenicity, or safety. Vaccine doses of 50 µg, 100 µg, and 200 µg were eligible. We excluded case reports, case series, single‐arm studies, observational designs, conference abstracts, animal studies, unpublished non‐peer‐reviewed data, duplicate publications, and review articles. When overlapping populations were identified, the most comprehensive and recent publication was retained. Only English‐language studies were included, without geographic restriction.

**FIGURE 1:**
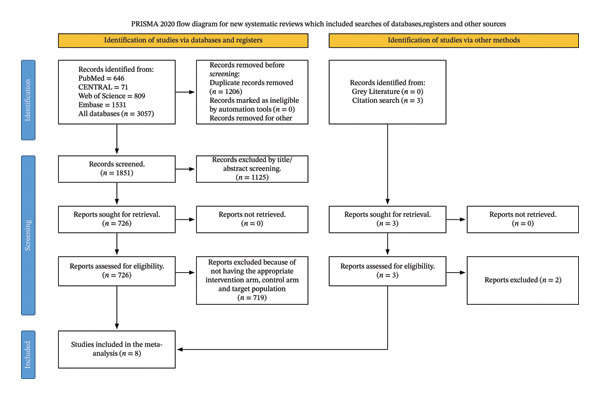
PRISMA 2020 flow diagram illustrating the systematic literature search, screening, eligibility assessment, and final study inclusion process. A total of 3057 records were identified across four databases (PubMed, Embase, Cochrane CENTRAL, Web of Science), with eight randomized controlled trials meeting inclusion criteria.

All records were imported into Mendeley (version 1.19.8) for duplicate removal. Two reviewers (MS and SA) independently screened titles and abstracts, followed by full‐text review for eligibility. Disagreements were resolved through discussion, with arbitration by a third reviewer (AJ) when necessary. The study selection process is illustrated using a PRISMA flow diagram (Figure 1). Data extraction was performed independently by two reviewers (MS and SA) using a predefined Excel template. Extracted data included study characteristics, participant demographics, intervention and comparator details, dosing regimens, follow‐up duration, and outcome data. Discrepancies were resolved by consensus or third‐reviewer adjudication. Data extraction followed the PICOS framework (population, intervention, comparator, outcomes, study design) [15].

The primary outcomes were vaccine efficacy, defined as prevention of symptomatic *C. difficile* infection, and immunogenicity, assessed by geometric mean concentrations of serum neutralizing antibodies against toxins A and B and by seroconversion rates. Secondary outcomes included vaccine safety and tolerability, encompassing local injection‐site reactions, systemic adverse events such as fever and fatigue, serious adverse events, and mortality. Vaccine doses of 50–100 µg and 200 µg were analyzed separately to evaluate dose‐dependent effects. To account for differences in dosing schedules, studies were stratified by day‐regimen and month‐regimen protocols. For day‐regimen studies, immunogenicity outcomes were extracted at Day 30. For month‐regimen studies, immunogenicity data were extracted at Month 7. This approach allowed standardized comparisons across trials with heterogeneous follow‐up schedules.

Risk of bias for individual studies was assessed independently using the Cochrane Risk of Bias 2.0 tool, evaluating randomization, deviations from intended interventions, missing outcome data, outcome measurement, and selective reporting. Each domain was categorized as low risk, some concerns, or high risk of bias. Certainty of evidence for each outcome was evaluated using the GRADE framework, considering study limitations, consistency, imprecision, indirectness, and publication bias [16].

Statistical analyses were conducted using RevMan version 5.4 (Cochrane Collaboration, Copenhagen, Denmark) with a random‐effects model to account for clinical heterogeneity. Dichotomous outcomes were summarized as relative risks with 95% confidence intervals, while continuous outcomes were analyzed using weighted mean differences with 95% confidence intervals. Statistical significance was defined as *p* < 0.05. Heterogeneity was assessed using the chi‐squared test and Higgins I^2^ statistic. Funnel plots were visually inspected for outcomes reported in more than 10 studies. For outcomes reported in fewer studies, DOI plots using the Luis Furuya–Kanamori index were generated in MetaXL to assess publication bias [17]. Sensitivity analyses were performed by excluding studies at high risk of bias and to explore sources of heterogeneity. Data synthesis followed guidance from the Cochrane Handbook and Rücker et al. [18].

## 3. Results

### 3.1. Study Selection and Included Studies

The literature search identified 3057 records, of which eight randomized controlled trials met the eligibility criteria and were included in the final analysis [19–26] (Figure 1). A total of 11,717 participants were included, comprising 7903 patients in the vaccine groups and 3814 patients in the placebo groups. Two studies were conducted in Japan [19, 20], five in the United States [21–25], and one multinational study across 27 countries [26]. Trials were published between 2016 and 2023 and included phase 1, phase 2, and phase 3 randomized, placebo‐controlled, observer‐blinded designs.

### 3.2. Participant Characteristics

Across all included studies, participant ages ranged from 50 to 85 years. Six studies evaluated day‐regimen vaccination schedules using 50 µg and 100 µg doses and included 9823 participants (6580 vaccine; 3243 placebo). The mean age was 71.6 ± 5.0 years in the vaccine group and 72.1 ± 5.0 years in the placebo group (Table 1).

**TABLE 1:** Baseline characteristics of participants in day‐regimen studies (50–100‐μg vaccine vs placebo).

Study ID	Conducted year	Study country	Study design	Participant age group (years)	Vaccine formulation	Follow‐up period	Total participants	Number of subjects in intervention (total toxoid A and B vaccine)	Number of subjects in control (normal saline placebo)	Age (years), mean(SD)		Male ratio		Female ratio	
										Vaccine	Placebo	Vaccine	Placebo	Vaccine	Placebo
De Bruyn	2021	27 countries (USA, Canada, Latin America, Europe, Asia‐pacific)	Phase 3 multicenter, observer‐blind, randomized, controlled trial	≥ 50	Toxoids A and B from *C. difficile*, combined with 400‐µg aluminum adjuvant per dose	3 years	9302	6201	3101	65.9 (8.9)	65.8 (8.9)	3556/6173	1796/3085	2617/6173	1289/3085
Inoue	2019	Japan	Phase 1, randomized, observer‐blinded, placebo‐controlled study	65–85	Genetically detoxified and chemically inactivated toxoids A and B, containing aluminum hydroxide	7 Months	22	14	8	72.7 (3.93)	73.0 (3.16)	5/14	3/8	9/14	5/8
Matsuoka	2018	Japan	Randomized, placebo‐controlled, Phase I/II study	40–75	Toxoids A and B, formaldehyde‐inactivated, from *C. difficile* strain ATCC 43255, with aluminum adjuvant	60 days	102	68	34	53.8 (9.5)	57.3 (9.5)	38/68	19/34	30/68	15/34
Remich	2020	USA	Phase 2, placebo‐controlled, randomized, observer‐blinded study	65–85	Genetically and chemically detoxified toxins A and B, combined with 1 mg/mL aluminum hydroxide	36 months	243	182	61	71.9 (5.27)	71.4 (4.89)	26 (42.6)	89 (48.9)	35 (57.4)	93 (51.1)
Lawrence	2021	USA and Canada	Phase 2, placebo‐controlled, randomized, observer‐blinded study	50–85	Investigational bivalent *Clostridioides difficile* vaccine, consisting of detoxified toxins A and B, containing aluminum hydroxide	12 months postdose 3 (day 30)	106	79	27	68.04 (4.59)	67.76 (4.87)	37/79	13/27	42/79	14/27
Greenberg	2012	USA (conducted at multiple centers)	Phase I, randomized, placebo‐controlled, double‐blind, dose‐ranging study	≥ 65 (elderly)	*C. difficile* toxoids A and B, adjuvanted with aluminum hydroxide, inactivated with formaldehyde	236 days	48	36	12	72 (6)	69 (4)	16/36	6/12	20/36	6/12

*Note:* µg, micrograms; Al(OH)_3_, aluminum hydroxide.

Abbreviations: ATCC, American Type Culture Collection; CDI, Clostridioides difficile infection; SD, standard deviation; USA, United States of America.

Four studies evaluated month‐regimen schedules using a 100‐µg dose (low dose) and included 352 participants (231 vaccine; 121 placebo). The mean age was 70.8 ± 4.2 years in the vaccine group and 69.9 ± 4.0 years in the placebo group (Table 2). Four studies evaluated month‐regimen schedules using a 200‐µg dose (high dose) and included 1542 participants (1092 vaccine; 450 placebo). The mean age was 63.2 ± 5.7 years in the vaccine group and 62.4 ± 4.3 years in the placebo group (Table 3).

**TABLE 2:** Baseline characteristics of participants in month‐regimen studies (100‐μg vaccine vs placebo).

Study ID	Conducted year	Study country	Study design	Participant age group (years)	Vaccine formulation	Follow‐up period	Total participants	Number of subjects in intervention (total toxoid A and B vaccine)	Number of subjects in control (normal saline placebo)	Age (years), mean(SD)		Female ratio		Male ratio	
										Vaccine	Placebo	Vaccine	Placebo	Vaccine	Placebo
Inoue	2019	Japan	Phase 1, randomized, observer‐blinded, placebo‐controlled study	65–85	Genetically detoxified and chemically inactivated toxoids A and B, containing aluminum hydroxide	12 months	40	24	16	68.8 (3.50)	69.9 (4.01)	10/24	9/16	14/24	7/16
Remich	2020	USA	Phase 2, placebo‐controlled, randomized, observer‐blinded study	65–85	Genetically and chemically detoxified toxins A and B, combined with 1 mg/mL aluminum hydroxide	36 months	244	183	61	70.4 (4.65)	71.5 (4.96)	37/61 (60.7)	93/183 (50.8)	24/61 (39.3)	90/183 (49.2)
Sheldon	2016	USA	Phase 1, placebo‐controlled, randomized, observer‐blinded study	50–85	Genetically detoxified and chemically inactivated toxoids A and B, containing aluminum hydroxide	12 months	68	24	44	61.8 (7.2)	62.4 (8.8)	16/24	24/44	8/24	20/44

*Note:* µg, micrograms; Al(OH)_3_, aluminum hydroxide.

Abbreviations: CDI, *Clostridioides difficile* infection; SD, standard deviation; USA, United States of America.

**TABLE 3:** Baseline characteristics of participants in month‐regimen studies (200‐μg vaccine vs placebo).

Study ID	Conducted year	Study country	Study design	Participant age group (years)	Vaccine formulation	Follow‐up period	Total participants	Number of subjects in intervention (total toxoid A and B vaccine)	Number of subjects in control (normal saline placebo)	Age (years), mean(SD)		Male ratio		Female ratio	
										Vaccine	Placebo	Vaccine	Placebo	Vaccine	Placebo
Christensen	2023	USA	Phase 3, randomized, observer‐blinded, placebo‐controlled study	65–85	Detoxified *C. difficile* toxins A and B, Adjuvant: Aluminum hydroxide (Al(OH)3)	7 months	1317	985	332	71.6 (4.96)	72.1 (4.99)	422/983	153/331	561/983	178/331
Inoue	2019	Japan	Phase 1, randomized, observer‐blinded, placebo‐controlled study	65–85	Genetically detoxified and chemically inactivated toxoids A and B, containing aluminum hydroxide	12 months	40	24	16	70.8 (4.18)	69.9 (4.01)	10/24	7/16	14/24	9/16
Remich	2020	USA	Phase 2, placebo‐controlled, randomized, observer‐blinded study	65–85	Genetically and chemically detoxified toxins A and B, combined with 1 mg/mL aluminum hydroxide	36 months	117	59	58	72.0 (4.53)	71.5 (4.81)	37/59	24/58	22/59	34/58
Sheldon	2016	USA	Phase 1, placebo‐controlled, randomized, observer‐blinded study	50–85	Genetically detoxified and chemically inactivated toxoids A and B, containing aluminum hydroxide	12 months	68	24	44	63.2 (5.68)	62.35 (4.25)	11/24	20/44	13/24	24/44
Donskey	2024	USA	Phase 3, placebo‐controlled, randomized observer‐blinded study	≥ 50	0.5 mL aluminum hydroxide‐containing PF‐06425090	12 months	17,440	8722	8718	68.0 (7.5)	68.1 (7.5)	4250/8722	4217/8718	4472/8722	4501/8718

*Note:* µg, micrograms; Al(OH)_3_, aluminum hydroxide; PF‐06425090, investigational *C. difficile* toxoid vaccine.

Abbreviations: CDI, *Clostridioides difficile* infection; SD, standard deviation; USA, United States of America.

#### 3.2.1. Risk of Bias and Quality Assessment of Outcomes

**FIGURE 2:**
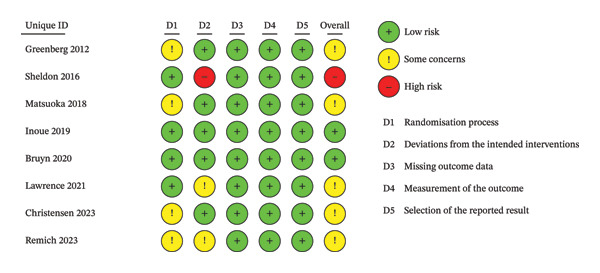
Risk of bias assessment for the eight included randomized controlled trials, evaluated using the Cochrane risk of bias 2.0 tool across five domains: randomization process, deviations from intended interventions, missing outcome data, measurement of the outcome, and selection of reported results. Each domain is categorized as low risk, some concerns, or high risk of bias.

Risk of bias assessment demonstrated that five studies were rated as low risk, two raised some concerns, and one was categorized as high risk (Figure 2). Certainty of evidence for each outcome was assessed using the GRADE framework, with detailed assessments provided in Supporting File 1.

#### 3.2.2. Primary Outcomes

##### 3.2.2.1. Vaccine Efficacy

###### 3.2.2.1.1. Postintervention Frequency of Overall CDI

**FIGURE 3:**
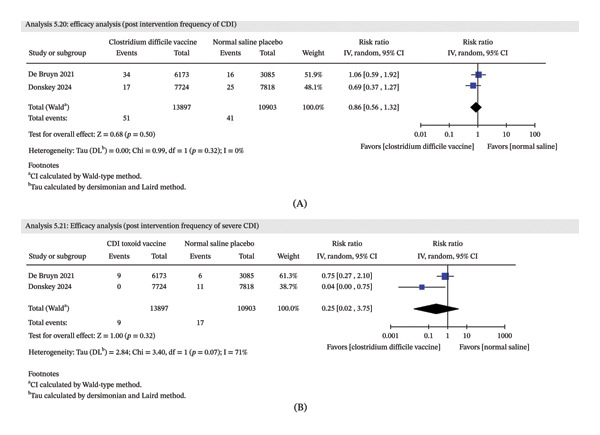
Forest plots comparing *Clostridioides difficile* toxoid vaccine versus placebo groups for (A) overall incidence of *Clostridioides difficile* infection (CDI) and (B) severe CDI after intervention. Effect estimates are expressed as relative risks (RRs) with 95% confidence intervals (CIs) using a random‐effects model. Heterogeneity is reported using the I‐squared statistic.

There was no significant difference in postintervention frequency of *Clostridium difficile* infection between the vaccine group and the placebo group (RR 0.86, 95% CI 0.56–1.32; *p* = 0.50). No heterogeneity was observed (I^2^ = 0%). The quality of evidence was rated as moderate due to imprecision (Figure 3).

###### 3.2.2.1.2. Postintervention Frequency of Severe CDI

Postintervention frequency of severe CDI did not differ significantly between groups (RR 0.25, 95% CI 0.02–3.75; *p* = 0.32). Heterogeneity was substantial (I^2^ = 71%). The quality of evidence was rated as moderate due to imprecision (Figure 3).

##### 3.2.2.2. Immunogenicity Outcomes

###### 3.2.2.2.1. Day‐Regimen Vaccine Studies

**FIGURE 4:**
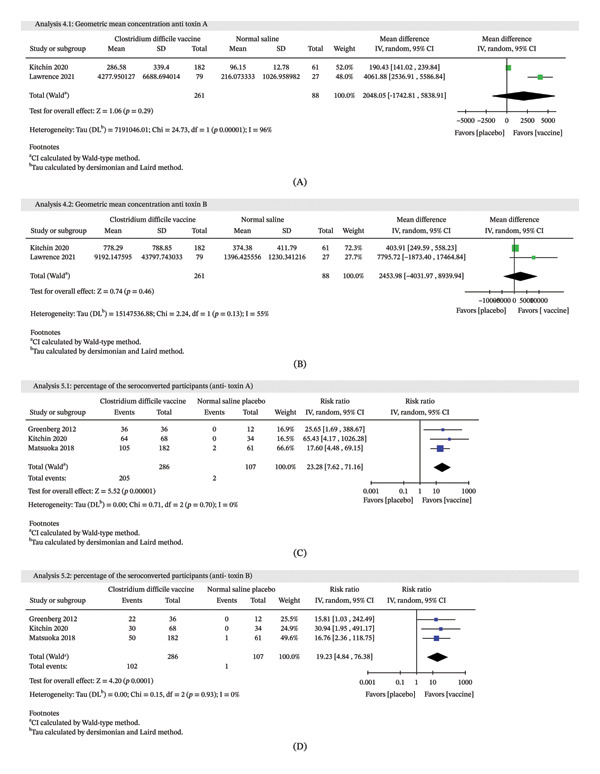
Forest plots for immunogenicity outcomes in day‐regimen studies (50–100‐μg doses), including (A) geometric mean concentration (GMC) of antitoxin A serum neutralizing antibodies, (B) GMC of antitoxin B serum neutralizing antibodies, (C) seroconversion rates against toxin A, and (D) seroconversion rates against toxin B. Effect estimates are expressed as mean differences (GMC) or relative risks (seroconversion) with 95% confidence intervals using a random‐effects model.

 Geometric Mean Concentration for Antitoxin A Serum Antibodies
 Two studies reported geometric mean concentrations of antitoxin A serum neutralizing antibodies. The pooled mean difference was 2048.05 units/mL (95% CI −1742.81 to 5838.91), with substantial heterogeneity (I^2^ = 96%). Evidence quality was low due to a serious risk of bias and imprecision (Figure 4).
 Geometric Mean Concentration for Antitoxin B Serum Antibodies
 Two studies reported geometric mean concentrations of antitoxin B serum antibodies. The pooled mean difference was 2453.98 units/mL (95% CI −4031.97 to 8939.94), with moderate heterogeneity (I^2^ = 55%). Evidence quality was low due to a serious risk of bias and imprecision (Figure 4).
 Seroconversion Rate Against Toxin A
 Three studies reported seroconversion rates against toxin A, which were higher in the vaccine group (RR 23.28, 95% CI 7.62–71.16). No heterogeneity was observed (I^2^ = 0%). Evidence quality was high (Figure 4).
 Seroconversion Rate Against Toxin B
 Three studies reported seroconversion rates against toxin B (RR 19.23, 95% CI 4.84–76.38). No heterogeneity was observed (I^2^ = 0%). Evidence quality was high (Figure 4).

###### 3.2.2.2.2. Month‐Regimen Vaccine Studies—100‐μg Dose (Low Dose)

**FIGURE 5:**
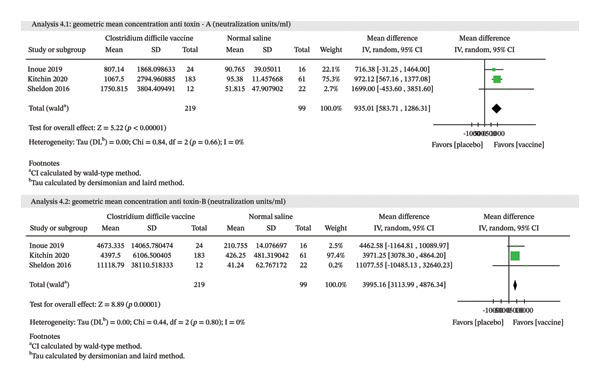
Forest plots for immunogenicity outcomes in month‐regimen studies receiving 100‐μg vaccine doses, showing geometric mean concentration (GMC) of antitoxin A and antitoxin B serum neutralizing antibodies. Effect estimates are expressed as mean differences (units/mL) with 95% confidence intervals using a random‐effects model.

 Geometric Mean Concentration for Antitoxin A Serum Antibodies
 Three studies reported significantly higher geometric mean concentrations of antitoxin A antibodies in the vaccine group (MD 935.01 units/mL, 95% CI 583.71–1286.31), with no heterogeneity (I^2^ = 0%). Evidence quality was moderate due to imprecision (Figure 5).
 Geometric Mean Concentration for Antitoxin B Serum Antibodies
 Three studies demonstrated higher geometric mean concentrations of antitoxin B antibodies in the vaccine group (MD 3995.16 units/mL, 95% CI 3113.99–4876.34), with no heterogeneity (I^2^ = 0%). Evidence quality was moderate due to imprecision (Figure 5).

###### 3.2.2.2.3. Month‐Regimen Vaccine Studies—200‐µg Dose (High Dose)

**FIGURE 6:**
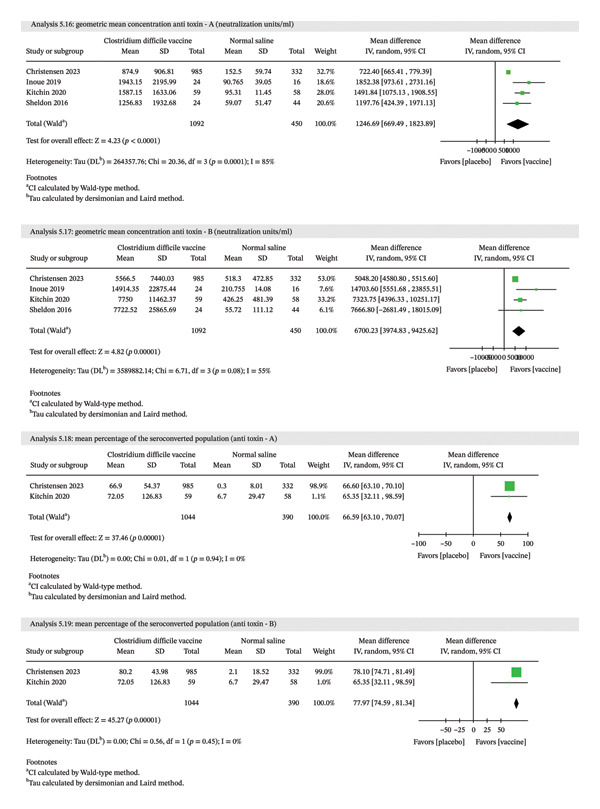
Forest plots for immunogenicity outcomes in month‐regimen studies receiving 200‐μg vaccine doses, including geometric mean concentrations (GMCs) of antitoxin A and antitoxin B serum neutralizing antibodies and mean percentage seroconversion for toxins A and B. Effect estimates are expressed as mean differences with 95% confidence intervals using a random‐effects model.

 Geometric Mean Concentration for Antitoxin A Serum Antibodies
 Four studies reported higher geometric mean concentrations of antitoxin A antibodies in the vaccine group (MD 1246.69 units/mL, 95% CI 669.49–1823.89), with substantial heterogeneity (I^2^ = 85%). Evidence quality was moderate due to inconsistency (Figure 6).
 Geometric Mean Concentration for Antitoxin B Serum Antibodies
 Four studies reported higher geometric mean concentrations of antitoxin B antibodies in the vaccine group (MD 6700.23 units/mL, 95% CI 3974.83–9425.62), with moderate heterogeneity (I^2^ = 55%). Evidence quality was moderate due to inconsistency (Figure 6).
 Seroconversion Rates
 Two studies reported higher seroconversion rates against toxin A (MD 66.59%, 95% CI 63.10–70.07; I^2^ = 0%) and toxin B (MD 77.97%, 95% CI 74.59–81.34; I^2^ = 0%). Evidence quality was moderate due to risk of bias (Figure 6).

#### 3.2.3. Secondary Outcomes

##### 3.2.3.1. Day‐Regimen Vaccine Studies

 Local Adverse Events
 Local adverse events were significantly more frequent in vaccine recipients, including pain (RR 3.02, 95% CI 2.47–3.70; I^2^ = 0%), swelling (RR 8.53, 95% CI 3.15–23.14; I^2^ = 0%), and erythema (RR 6.78, 95% CI 2.35–19.61; I^2^ = 0%) (Supporting Figure 1).
 Systemic Adverse Events and Mortality
 Systemic adverse events demonstrated mixed results. Myalgia was increased in the vaccine group (RR 1.26, 95% CI 1.06–1.50; I^2^ = 0%), whereas fever (RR 0.85, 95% CI 0.56–1.29; I^2^ = 0%) and malaise or fatigue (RR 1.15, 95% CI 0.74–1.80; I^2^ = 43%) showed no significant differences between groups. Similarly, headache (RR 1.11, 95% CI 0.93–1.32; I^2^ = 0%) and arthralgia (RR 1.16, 95% CI 0.94–1.43; I^2^ = 0%) showed no significant differences (Supporting Figure 2). No significant associations were observed for infections or infestations (RR 1.06, 95% CI 0.95–1.18; I^2^ = 0%) or musculoskeletal disorders (RR 1.05, 95% CI 0.89–1.23; I^2^ = 0%). Gastrointestinal disorders demonstrated a trend toward reduced risk, although heterogeneity was substantial (RR 0.54, 95% CI 0.24–1.19; I^2^ = 72%). Overall adverse events (RR 2.00, 95% CI 0.30–13.54; I^2^ = 55%) and serious adverse events (RR 0.98, 95% CI 0.91–1.05; I^2^ = 0%) were not significantly different between groups. Mortality was nonsignificantly lower in the vaccine group (RR 0.34, 95% CI 0.05–2.38) (Supporting Figures 1–4).

##### 3.2.3.2. Month‐Regimen Vaccine Studies—100‐µg Dose (Low Dose)

 Local Adverse Events
 Local adverse events were significantly increased in the vaccine group, including pain (RR 5.39, 95% CI 2.06–14.11; I^2^ = 26%), swelling (RR 4.91, 95% CI 1.63–14.78; I^2^ = 0%), and erythema (RR 4.17, 95% CI 1.41–12.33; I^2^ = 0%) (Supporting Figure 5).
 Systemic Adverse Events
 Systemic adverse events showed mixed findings. Myalgia (RR 1.14, 95% CI 0.75–1.73; I^2^ = 0%) and headache (RR 2.80, 95% CI 0.19–41.41; I^2^ = 95%) were more frequent in the vaccine group, whereas malaise or fatigue (RR 0.93, 95% CI 0.40–2.16; I^2^ = 27%) and arthralgia (RR 1.00, 95% CI 0.52–1.92; I^2^ = 0%) showed no significant difference between groups. The vaccine group demonstrated a lower rate of infections and infestations (RR 0.75, 95% CI 0.12–4.90; I^2^ = 83%). Gastrointestinal disorders showed a trend toward higher risk with low heterogeneity but low certainty of evidence (RR 1.43, 95% CI 0.74–2.76; I^2^ = 0%). Additionally, lower risks of skin or subcutaneous tissue disorders (RR 0.52, 95% CI 0.11–2.44; I^2^ = 46%) and musculoskeletal disorders (RR 0.75, 95% CI 0.28–2.03) were observed, while respiratory or thoracic disorders occurred more frequently in the vaccine group (RR 1.06, 95% CI 0.59–1.88). No significant differences were observed in overall adverse events or serious adverse events between groups (RR 1.03, 95% CI 0.87–1.22) (Supporting Figures 5–7).

##### 3.2.3.3. Month‐Regimen Vaccine Studies—200‐µg Dose (High Dose)

 Local Adverse Events
 Local adverse events were significantly increased in the vaccine group, including pain (RR 8.18, 95% CI 2.28–29.38; I^2^ = 77%), swelling (RR 6.44, 95% CI 5.52–7.52; I^2^ = 0%), and erythema (RR 4.93, 95% CI 4.21–5.78; I^2^ = 0%) (Supporting Figure 8).
 Systemic Adverse Events
 Systemic adverse events demonstrated mixed results. Fatigue or malaise (RR 1.20, 95% CI 0.94–1.53; I^2^ = 41%) and arthralgia (RR 1.16, 95% CI 0.83–1.60; I^2^ = 28%) were increased in the vaccine group, while myalgia occurred less frequently (RR 1.10, 95% CI 0.80–1.51; I^2^ = 31%). Headache showed no significant difference between groups (RR 1.24, 95% CI 0.92–1.65; I^2^ = 49%). No significant differences were observed between groups for infections or infestations (RR 0.46, 95% CI 0.14–1.52; I^2^ = 29%), gastrointestinal disorders (RR 1.00, 95% CI 0.82–1.23; I^2^ = 0%), or respiratory or thoracic disorders (RR 1.01, 95% CI 0.64–1.59; I^2^ = 1%). The vaccine group experienced fewer skin or subcutaneous tissue infections (RR 1.06, 95% CI 0.05–21.6) and more musculoskeletal disorders (RR 2.55, 95% CI 0.96–6.81; I^2^ = 0%). There were no significant differences between groups in serious adverse events (RR 1.02, 95% CI 0.95–1.09; I^2^ = 0%) or overall adverse events (RR 1.02, 95% CI 0.99–1.05; I^2^ = 0%). Mortality was lower in the vaccine group, although this difference was not statistically significant (RR 0.92, 95% CI 0.42–2.02; I^2^ = 26%) (Supporting Figures 8–11).

## 4. Discussion

The burden of CDI and its associated morbidity and mortality continue to create a strong rationale for preventive strategies, including vaccination [27]. In this systematic review and meta‐analysis of randomized controlled trials, *C. difficile* toxoid vaccination demonstrated a consistent immunogenic response and an acceptable overall safety profile but did not show a statistically significant reduction in the overall CDI incidence. Collectively, these findings suggest that current toxoid vaccine approaches reliably induce toxin‐directed humoral immunity, yet the translation of immunogenicity into clinically meaningful protection remains uncertain.

A central observation across included trials was the vaccine’s reactogenicity profile. Local adverse events, including pain, swelling, and erythema, occurred more frequently among vaccine recipients across dosing schedules and dose levels. This pattern aligns with prior reports from clinical development studies evaluating toxoid vaccines administered with and without aluminum adjuvant [21–23]. Differences in local reaction rates across formulations have been reported, including higher reactogenicity with toxoid‐only formulations relative to toxoid‐alum formulations in some studies and the inverse in others, indicating that both antigen content and adjuvant strategy may influence tolerability [28]. Importantly, while local reactions were common, the overall evidence did not demonstrate a consistent increase in serious adverse events.

Systemic adverse events were generally comparable between vaccine and placebo groups. Across day‐regimen and month‐regimen protocols, systemic symptoms such as fever and malaise or fatigue were not consistently elevated, although some dose‐dependent increases in transient systemic symptoms were noted in individual trials [19, 20]. These findings support that the toxoid vaccine strategy is broadly tolerable in older adult populations, which is essential for any preventive intervention targeting those at highest CDI risk.

Seroconversion analyses demonstrated higher rates in vaccine recipients than in placebo recipients with no observed heterogeneity (I^2^ = 0%), supporting confidence in the direction and magnitude of these pooled estimates. By contrast, geometric mean concentration analyses showed substantial heterogeneity (I^2^ up to 96%); while the direction of effect favored vaccination across regimens, the precision of any dose–response inference is limited, as observed between‐study variation may reflect methodological differences rather than true biological dose effects. Heterogeneity in immunogenicity reporting and measurement units also constrained harmonization across all studies, limiting the ability to establish a quantitative relationship between antibody magnitude and clinical outcomes.

Despite strong immunogenicity, vaccine efficacy against overall CDI incidence was not statistically significant. This finding is consistent with large randomized trials evaluating toxoid‐based candidates [26, 29]. Several explanations may account for the observed dissociation between antibody response and incident CDI. First, toxoid vaccines target toxin‐mediated pathology rather than colonization and therefore may be less likely to prevent infection acquisition. Second, immunosenescence and comorbidity burden in older adults may influence antibody function and durability, potentially reducing clinical effectiveness despite measurable serologic responses. Third, outcome definitions and case ascertainment varied across trials, and CDI incidence may be driven by exposures and host factors not fully modifiable through toxin neutralization alone.

A clinically relevant question is whether vaccination may reduce disease severity even if it does not prevent infection. While our analysis did not demonstrate a statistically significant reduction in severe CDI incidence and was limited by substantial heterogeneity and a small number of contributing studies, the point estimate suggested a potential protective signal. This possibility is supported by secondary analyses reported in large clinical trials, including CLOVER, which did not meet its primary endpoint, with a vaccine efficacy of 31% (96.4% CI −38.7% to 66.6%) for overall CDI, but reported a post hoc vaccine efficacy of 100% (95% CI 59.6–100%) for medically attended CDI [30]. The dissociation between immunogenicity, overall efficacy, and severity‐stratified efficacy, combined with substantial heterogeneity in antibody concentration outcomes, complicates the identification of a serologic correlate of protection threshold and supports prioritization of severity‐based endpoints in future trial design. These findings raise the possibility that toxoid vaccination could shift the clinical phenotype toward less severe disease, although this hypothesis requires confirmation in trials specifically designed and powered for severity endpoints with standardized definitions.

This study has several strengths. We included only randomized controlled trials, minimizing confounding and selection bias, and analyzed a large cumulative sample size spanning multiple trial phases and geographic settings. However, important limitations should be considered. First, immunogenicity outcomes could not be pooled across all studies due to inconsistent reporting units and heterogeneous assay approaches, which limits cross‐trial comparability. Second, the number of trials contributing to severe CDI outcomes was small, and heterogeneity was substantial, resulting in imprecise estimates. Third, variation in vaccine formulations and regimens, including evaluation of a toxoid formulation without aluminum adjuvant in one trial and differences in dose and participant demographics across studies, may contribute to clinical and statistical heterogeneity [24, 25]. Fourth, heterogeneity was particularly high in immunogenicity analyses for geometric mean concentration outcomes (I^2^ = 96%, *τ* ^2^ = 7,191,046 for antitoxin A in day‐regimen studies; I^2^ = 85%, *τ* ^2^ = 264,358 for antitoxin A in 200‐μg month‐regimen studies), likely reflecting differences in immunoassay methodology, antigen preparation, adjuvant content, and participant age distributions across trials. Interlaboratory variation of several‐fold has been documented for standardized vaccine immunoassays even when testing identical sera, supporting assay methodology as a plausible contributor to between‐study variance [31]. For the 200‐μg month‐regimen analyses, 95% prediction intervals were calculable and were wide enough to cross the null (antitoxin A: −1303 to 3796 units/mL; antitoxin B: −3412 to 16,812 units/mL), indicating that the expected range of true effects in future studies is consistent with both substantial benefit and no benefit. Prediction intervals were not computed for day‐regimen geometric mean concentration analyses because only two studies contributed to each, which is below the minimum number generally recommended for stable estimation. Formal meta‐regression to test sources of heterogeneity was precluded by the small number of contributing studies per outcome (*k* = 2–4), which would yield unstable coefficient estimates. This degree of between‐study variation substantially limits confidence in establishing a dose–response gradient from pooled estimates, as observed differences in antibody magnitude may reflect methodological heterogeneity rather than true biological effects of dose. Pooled estimates from these analyses should therefore be interpreted as indicative of the direction of effect rather than as precise quantitative summaries. By contrast, where heterogeneity was absent, as in seroconversion rate analyses (I^2^ = 0%), greater confidence in the pooled estimates is warranted. Finally, restriction to English‐language studies may introduce language bias.

Future research should focus on clarifying immunologic correlates of protection, including functional toxin‐neutralizing activity and durability of responses in older adults, and should prioritize standardized outcome definitions to improve comparability across trials. Given the potential for severity mitigation, adequately powered trials using clinically meaningful severity endpoints and healthcare utilization outcomes may be more informative than incidence‐only designs. At present, no toxoid vaccine has been approved for CDI prevention, and current evidence does not support clinical implementation outside of research settings.

## 5. Conclusion

This systematic review and meta‐analysis demonstrates that *Clostridioides difficile* toxoid vaccines consistently elicit strong toxin‐specific humoral immune responses and are associated with an acceptable safety profile, with adverse effects largely limited to transient local reactogenicity. Across randomized controlled trials, vaccination did not result in a statistically significant reduction in overall CDI incidence; however, immunogenicity outcomes were robust and reproducible across dosing regimens and study phases. Although evidence for reduction in severe CDI remains limited and heterogeneous, the direction of effect observed across studies, together with findings from large phase 3 trials, suggests a potential role for toxoid vaccination in mitigating disease severity rather than preventing infection. Future studies should focus on defining immunologic correlates of protection, standardizing severity‐based clinical endpoints, and clarifying whether toxin‐directed immunity can translate into meaningful reductions in severe disease and healthcare utilization [32].

## Author Contributions

• Muhammad Shahzil: conceptualization, methodology, data curation, formal analysis, writing–original draft, critical revision, supervision, and project administration.

• Zainab Jamil: data collection, literature review, data interpretation, writing–review and editing, and critical revision.

• Saleha Azeem: data collection, literature review, data interpretation, writing–review and editing, and critical revision.

• Minahel Shehzadi: data collection, literature review, data interpretation, writing–review and editing, and critical revision.

• Aisha Shabbir: data curation, data analysis, critical interpretation, and writing–review and editing.

• Muhammad Saad Faisal: data analysis, visualization, statistical interpretation, writing–review and editing, and critical revision.

• Ammad Javaid Chaudhary: tatistical analysis, data interpretation, writing–review and editing, and critical revision.

• Fariha Hasan: literature search, data validation, data interpretation, and writing–review and editing.

• Muhammad Ali Khaqan: Methodological input, data interpretation, critical review, writing–review and editing.

• Syed‐Mohammed Jafri: supervision, critical interpretation of results, writing–review and editing, and final approval of manuscript.

## Funding

(i) Article processing charges for open access publishing are covered through the affiliation with the Pennsylvania State University. No other funding was received for the research and authorship of this article.

(ii) The writing and preparation of this paper were not funded by any organization.

(iii) Initial data analyses were undertaken by the authors themselves and did not receive any external funding.

(iv) No external writing support was provided for this paper.

## Ethics Statement

The authors have nothing to report.

## Consent

The authors have nothing to report.

## Conflicts of Interest

The authors declare no conflicts of interest.

## Supporting Information

Additional supporting information can be found online in the Supporting Information section.

## Supporting information


**Supporting Information 1** Supporting File 1 includes the full electronic database search strategies used for systematic literature identification, comprehensive GRADE evidence profiles for each outcome, and expanded forest plots for local, systemic, and overall adverse events categorized by vaccine regimen and dose.

**Supporting Information 2** Supporting Figure 1. Forest plots for local adverse events (pain, swelling, erythema) in day‐regimen studies comparing *Clostridioides difficile* toxoid vaccine versus placebo groups. Effect estimates are expressed as relative risks (RRs) with 95% confidence intervals (CIs) using a random‐effects model. Heterogeneity is reported using the I‐squared statistic.

**Supporting Information 3** Supporting Figure 2. Forest plots for systemic adverse events (fever, fatigue/malaise, myalgia, headache, arthralgia) in day‐regimen studies comparing vaccine versus placebo groups. Effect estimates are expressed as RR with 95% CI using a random‐effects model.

**Supporting Information 4** Supporting Figure 3. Forest plots for overall adverse events, serious adverse events, and mortality in day‐regimen studies comparing vaccine versus placebo groups. Effect estimates are expressed as RR with 95% CI using a random‐effects model.

**Supporting Information 5** Supporting Figure 4. Forest plots for infections/infestations, gastrointestinal disorders, musculoskeletal complaints, and respiratory disorders in day‐regimen studies. Effect estimates are expressed as RR with 95% CI using a random‐effects model.

**Supporting Information 6** Supporting Figure 5. Forest plots for local adverse events (pain, swelling, erythema) in month‐regimen studies receiving 100‐μg vaccine doses versus placebo. Effect estimates are expressed as RR with 95% CI using a random‐effects model.

**Supporting Information 7** Supporting Figure 6. Forest plots for systemic adverse events (fatigue, myalgia, headache, malaise, arthralgia) in month‐regimen studies receiving 100‐μg vaccine doses. Effect estimates are expressed as RR with 95% CI using a random‐effects model.

**Supporting Information 8** Supporting Figure 7. Forest plots for overall adverse events and serious adverse events in month‐regimen studies receiving 100‐μg vaccine doses. Effect estimates are expressed as RR with 95% CI using a random‐effects model.

**Supporting Information 9** Supporting Figure 8. Forest plots for local adverse events (pain, swelling, erythema) in month‐regimen studies receiving 200‐μg vaccine doses versus placebo. Effect estimates are expressed as RR with 95% CI using a random‐effects model.

**Supporting Information 10** Supporting Figure 9. Forest plots for systemic adverse events (fatigue, myalgia, headache, malaise, arthralgia) in month‐regimen studies receiving 200‐μg vaccine doses. Effect estimates are expressed as RR with 95% CI using a random‐effects model.

**Supporting Information 11** Supporting Figure 10. Forest plots for infections, gastrointestinal disorders, musculoskeletal issues, and respiratory disorders in month‐regimen studies receiving 200‐μg vaccine doses. Effect estimates are expressed as RR with 95% CI using a random‐effects model.

**Supporting Information 12** Supporting Figure 11. Forest plots for overall adverse events, serious adverse events, and mortality in month‐regimen studies receiving 200‐μg vaccine doses. Effect estimates are expressed as RR with 95% CI using a random‐effects model.

**Supporting Information 13** PRISMA 2020 Checklist.

## Data Availability

The datasets utilized and analyzed in this study are available in the Supporting file.
