# Supplementary material for: Comprehensive Evaluation of the Efficacy and Safety of the Clostridioides difficile Toxoid Vaccine: A Meta‐Analysis
Source: Can J Infect Dis Med Microbiol. 2026 Jul 30;2026:1160340. doi: 10.1155/cjid/1160340 (PMC13422635; doi:10.1155/cjid/1160340)
Supplement: Supplementary file 1 — Supporting Information 1 Supporting File 1 includes the full electronic database search strategies used for systematic literature identification, comprehensive GRADE evidence profiles for each outcome, and expanded forest plots for local, systemic, and overall adverse events categorized by vaccine regimen and dose. [file CJID-2026-1160340-s012.docx]

## **Search Strategy:**

PubMed:

|  | ("*Clostridium Difficile*" OR "C diff*" OR "*C. difficile*" OR "*Clostridium Difficile* infection*" OR "C diff infection*" OR "*C. Difficile* infection*" OR "*Clostridioides difficile*") |
| --- | --- |
|  | ("vaccin*" OR "vaccine" OR "vaccination") |
|  | #1 AND #2 |

Embase:

|  | ("*Clostridium Difficile*" OR "C diff*" OR "*C. difficile*" OR "*Clostridium Difficile* infection*" OR "C diff infection*" OR "*C. Difficile* infection*" OR "*Clostridioides difficile*") |
| --- | --- |
|  | ("vaccin*" OR "vaccine" OR "vaccination") |
|  | #1 AND #2 |

Cochrane:

|  | ("*Clostridium Difficile*" OR "C diff*" OR "*C. difficile*" OR "*Clostridium Difficile* infection*" OR "C diff infection*" OR "*C. Difficile* infection*" OR "*Clostridioides difficile*") |
| --- | --- |
|  | ("vaccin*" OR "vaccine" OR "vaccination") |
|  | #1 AND #2 |

Web of Science:

|  | ("*Clostridium Difficile*" OR "C diff*" OR "*C. difficile*" OR "*Clostridium Difficile* infection*" OR "C diff infection*" OR "*C. Difficile* infection*" OR "*Clostridioides difficile*") |
| --- | --- |
|  | ("vaccin*" OR "vaccine" OR "vaccination") |
|  | #1 AND #2 |

# GRADE Assessment:

## Day Regimen:

| Certainty assessment | | | | | | | № of patients | | Effect | | Certainty | Importance |
| --- | --- | --- | --- | --- | --- | --- | --- | --- | --- | --- | --- | --- |
| № of studies | Study design | Risk of bias | Inconsistency | Indirectness | Imprecision | Other considerations | Injection Site Reactions | [placebo] | Relative (95% CI) | Absolute (95% CI) |  |  |
| Pain | | | | | | | | | | | | |
| 6 | randomised trials | serious | not serious | not serious | not serious | publication bias strongly suspected strong association | 604/1578 (38.3%) | 90/728 (12.4%) | RR 3.02 (2.47 to 3.70) | 250 more per 1,000 (from 182 more to 334 more) | ⨁⨁⨁◯ Moderate |  |
| Swelling | | | | | | | | | | | | |
| 5 | randomised trials | serious | not serious | not serious | not serious | publication bias strongly suspected very strong association | 75/1540 (4.9%) | 2/717 (0.3%) | RR 8.53 (3.15 to 23.14) | 21 more per 1,000 (from 6 more to 62 more) | ⨁⨁⨁⨁ High |  |
| Erythema | | | | | | | | | | | | |
| 6 | randomised trials | serious | not serious | not serious | not serious | publication bias strongly suspected very strong association | 88/1575 (5.6%) | 1/728 (0.1%) | RR 6.78 (2.35 to 19.61) | 8 more per 1,000 (from 2 more to 26 more) | ⨁⨁⨁⨁ High |  |

| Fever | | | | | | | | | | | | |
| --- | --- | --- | --- | --- | --- | --- | --- | --- | --- | --- | --- | --- |
| 2 | randomised trials | not serious | not serious | not serious | serious | none | 58/1241 (4.7%) | 33/611 (5.4%) | RR 0.85 (0.56 to 1.29) | 8 fewer per 1,000 (from 24 fewer to 16 more) | ⨁⨁⨁◯ Moderate |  |
| Malaise/Fatigue | | | | | | | | | | | | |
| 5 | randomised trials | serious | not serious | not serious | not serious | none | 345/1561 (22.1%) | 134/720 (18.6%) | RR 1.15 (0.74 to 1.80) | 28 more per 1,000 (from 48 fewer to 149 more) | ⨁⨁⨁◯ Moderate |  |
| Myalgia | | | | | | | | | | | | |
| 6 | randomised trials | serious | not serious | not serious | serious | none | 355/1575 (22.5%) | 134/728 (18.4%) | RR 1.26 (1.06 to 1.50) | 48 more per 1,000 (from 11 more to 92 more) | ⨁⨁◯◯ Low |  |
| Headache | | | | | | | | | | | | |
| 4 | randomised trials | serious | not serious | not serious | not serious | none | 337/1525 (22.1%) | 144/708 (20.3%) | RR 1.11 (0.93 to 1.32) | 22 more per 1,000 (from 14 fewer to 65 more) | ⨁⨁⨁◯ Moderate |  |
| Arthralgia | | | | | | | | | | | | |
| 5 | randomised trials | not serious | not serious | not serious | not serious | none | 261/1561 (16.7%) | 104/720 (14.4%) | RR 1.16 (0.94 to 1.43) | 23 more per 1,000 (from 9 fewer to 62 more) | ⨁⨁⨁⨁ High |  |

| Infections and Infestations | | | | | | | | | | | | |
| --- | --- | --- | --- | --- | --- | --- | --- | --- | --- | --- | --- | --- |
| 2 | randomised trials | not serious | not serious | not serious | serious | none | 893/6181 (14.4%) | 420/3091 (13.6%) | RR 1.06 (0.95 to 1.18) | 8 more per 1,000 (from 7 fewer to 24 more) | ⨁⨁⨁◯ Moderate |  |
| GI | | | | | | | | | | | | |
| 4 | randomised trials | serious | serious | not serious | serious | none | 719/6410 (11.2%) | 392/3157 (12.4%) | RR 0.54 (0.24 to 1.19) | 57 fewer per 1,000 (from 94 fewer to 24 more) | ⨁◯◯◯ Very low |  |
| MSK | | | | | | | | | | | | |
| 3 | randomised trials | serious | not serious | not serious | not serious | none | 429/6217 (6.9%) | 203/3103 (6.5%) | RR 1.05 (0.89 to 1.23) | 3 more per 1,000 (from 7 fewer to 15 more) | ⨁⨁⨁◯ Moderate |  |
| Injury and Poisoning | | | | | | | | | | | | |
| 2 | randomised trials | not serious | not serious | not serious | serious | none | 310/6181 (5.0%) | 138/3091 (4.5%) | RR 1.12 (0.92 to 1.36) | 5 more per 1,000 (from 4 fewer to 16 more) | ⨁⨁⨁◯ Moderate |  |
| Serious Adverse Events | | | | | | | | | | | | |
| 2 | randomised trials | serious | not serious | not serious | serious | none | 1664/6131 (27.1%) | 852/3063 (27.8%) | RR 0.98 (0.91 to 1.05) | 6 fewer per 1,000 (from 25 fewer to 14 more) | ⨁⨁◯◯ Low |  |
| Adverse Events | | | | | | | | | | | | |
| 2 | randomised trials | not serious | serious | not serious | serious | strong association | 35/93 (37.6%) | 9/35 (25.7%) | RR 2.00 (0.30 to 13.54) | 257 more per 1,000 (from 180 fewer to 1,000 more) | ⨁⨁⨁◯ Moderate |  |

| Geometric Mean Concentration Anti Toxin A | | | | | | | | | | | | |
| --- | --- | --- | --- | --- | --- | --- | --- | --- | --- | --- | --- | --- |
| 2 | randomised trials | serious | not serious | not serious | serious | none | 261 | 88 | - | MD 2048.05 higher (1742.81 lower to 5838.91 higher) | ⨁⨁◯◯ Low |  |
| Geometric Mean Concentration Anti Toxin B | | | | | | | | | | | | |
| 2 | randomised trials | serious | not serious | not serious | serious | none | 261 | 88 | - | MD 2453.98 higher (4031.97 lower to 8939.94 higher) | ⨁⨁◯◯ Low |  |

| Percentage of the seroconverted participants (Anti- Toxin A) | | | | | | | | | | | | |
| --- | --- | --- | --- | --- | --- | --- | --- | --- | --- | --- | --- | --- |
| 3 | randomised trials | not serious | not serious | not serious | not serious | none | 205/286 (71.7%) | 2/107 (1.9%) | RR 23.28 (7.62 to 71.16) | 416 more per 1,000 (from 124 more to 1,000 more) | ⨁⨁⨁⨁ High |  |
| Percentage of the seroconverted participants (Anti- Toxin B) | | | | | | | | | | | | |
| 3 | randomised trials | not serious | not serious | not serious | not serious | none | 102/286 (35.7%) | 1/107 (0.9%) | RR 19.23 (4.84 to 76.38) | 170 more per 1,000 (from 36 more to 704 more) | ⨁⨁⨁⨁ High |  |

CI: confidence interval; RR: risk ratio

## Month Regimen- Low Dose (50-100ug)

| Certainty assessment | | | | | | | № of patients | | Effect | | Certainty | Importance |
| --- | --- | --- | --- | --- | --- | --- | --- | --- | --- | --- | --- | --- |
| № of studies | Study design | Risk of bias | Inconsistency | Indirectness | Imprecision | Other considerations | Injection Site Reactions | [placebo] | Relative (95% CI) | Absolute (95% CI) |  |  |
| Pain | | | | | | | | | | | | |
| 4 | randomised trials | not serious | serious | not serious | not serious | publication bias strongly suspected very strong association | 268/1214 (22.1%) | 16/452 (3.5%) | RR 5.39 (2.06 to 14.11) | 155 more per 1,000 (from 38 more to 464 more) | ⨁⨁⨁⨁ High |  |
| Swelling | | | | | | | | | | | | |
| 4 | randomised trials | not serious | not serious | not serious | not serious | publication bias strongly suspected strong association | 52/1214 (4.3%) | 3/452 (0.7%) | RR 4.91 (1.63 to 14.78) | 26 more per 1,000 (from 4 more to 91 more) | ⨁⨁⨁⨁ High |  |
| Erythema | | | | | | | | | | | | |
| 4 | randomised trials | not serious | not serious | not serious | not serious | strong association | 43/1214 (3.5%) | 3/452 (0.7%) | RR 4.17 (1.41 to 12.33) | 21 more per 1,000 (from 3 more to 75 more) | ⨁⨁⨁⨁ High |  |

| Malaise/Fatigue | | | | | | | | | | | | |
| --- | --- | --- | --- | --- | --- | --- | --- | --- | --- | --- | --- | --- |
| 3 | randomised trials | not serious | not serious | not serious | serious | none | 43/1190 (3.6%) | 14/436 (3.2%) | RR 0.93 (0.40 to 2.16) | 2 fewer per 1,000 (from 19 fewer to 37 more) | ⨁⨁⨁◯ Moderate |  |
| Myalgia | | | | | | | | | | | | |
| 3 | randomised trials | not serious | not serious | not serious | not serious | none | 91/1190 (7.6%) | 27/436 (6.2%) | RR 1.14 (0.75 to 1.73) | 9 more per 1,000 (from 15 fewer to 45 more) | ⨁⨁⨁⨁ High |  |
| Headache | | | | | | | | | | | | |
| 2 | randomised trials | serious | not serious | serious | serious | strong association | 159/1166 (13.6%) | 15/392 (3.8%) | RR 2.80 (0.19 to 41.41) | 69 more per 1,000 (from 31 fewer to 1,000 more) | ⨁⨁⨁◯ Moderate |  |
| Arthralgia | | | | | | | | | | | | |
| 2 | randomised trials | not serious | not serious | serious | serious | none | 33/1166 (2.8%) | 11/392 (2.8%) | RR 1.00 (0.52 to 1.92) | 0 fewer per 1,000 (from 13 fewer to 26 more) | ⨁⨁◯◯ Low |  |

| Infections and Infestations | | | | | | | | | | | | |
| --- | --- | --- | --- | --- | --- | --- | --- | --- | --- | --- | --- | --- |
| 2 | randomised trials | serious | not serious | not serious | serious | none | 12/1007 (1.2%) | 13/375 (3.5%) | RR 0.75 (0.12 to 4.90) | 9 fewer per 1,000 (from 31 fewer to 135 more) | ⨁⨁◯◯ Low |  |
| GI | | | | | | | | | | | | |
| 3 | randomised trials | not serious | not serious | not serious | not serious | none | 41/1190 (3.4%) | 11/436 (2.5%) | RR 1.43 (0.74 to 2.76) | 11 more per 1,000 (from 7 fewer to 44 more) | ⨁⨁⨁⨁ High |  |
| Skin | | | | | | | | | | | | |
| 2 | randomised trials | serious | not serious | not serious | serious | none | 5/1007 (0.5%) | 7/375 (1.9%) | RR 0.52 (0.11 to 2.44) | 9 fewer per 1,000 (from 17 fewer to 27 more) | ⨁⨁◯◯ Low |  |
| Respiratory | | | | | | | | | | | | |
| 2 | randomised trials | not serious | not serious | not serious | not serious | none | 42/1007 (4.2%) | 16/375 (4.3%) | RR 1.06 (0.59 to 1.88) | 3 more per 1,000 (from 17 fewer to 38 more) | ⨁⨁⨁⨁ High |  |
| MSK | | | | | | | | | | | | |
| 2 | randomised trials | not serious | not serious | not serious | not serious | none | 12/1007 (1.2%) | 9/375 (2.4%) | RR 0.75 (0.28 to 2.03) | 6 fewer per 1,000 (from 17 fewer to 25 more) | ⨁⨁⨁⨁ High |  |
| Injury and Poisoning | | | | | | | | | | | | |
| 2 | randomised trials | not serious | not serious | not serious | not serious | none | 15/1007 (1.5%) | 3/375 (0.8%) | RR 2.77 (0.80 to 9.52) | 14 more per 1,000 (from 2 fewer to 68 more) | ⨁⨁⨁⨁ High |  |
| Adverse Events | | | | | | | | | | | | |
| 2 | randomised trials | not serious | not serious | not serious | not serious | strong association | 363/1007 (36.0%) | 120/347 (34.6%) | RR 1.03 (0.87 to 1.22) | 10 more per 1,000 (from 45 fewer to 76 more) | ⨁⨁⨁⨁ High |  |
| Mortality | | | | | | | | | | | | |
| 2 | randomised trials | not serious | serious | not serious | not serious | none | 2/1007 (0.2%) | 2/347 (0.6%) | RR 0.34 (0.05 to 2.38) | 4 fewer per 1,000 (from 5 fewer to 8 more) | ⨁⨁⨁◯ Moderate |  |

| Geometric Mean Concentration Anti Toxin - A (Neutralization units/ml) | | | | | | | | | | | | |
| --- | --- | --- | --- | --- | --- | --- | --- | --- | --- | --- | --- | --- |
| 3 | randomised trials | not serious | not serious | not serious | serious | none | 219 | 99 | - | MD 935.01 higher (583.71 higher to 1286.31 higher) | ⨁⨁⨁◯ Moderate |  |
| Geometric Mean Concentration Anti Toxin - B (Neutralization units/ml) | | | | | | | | | | | | |
| 3 | randomised trials | not serious | not serious | not serious | serious | none | 219 | 99 | - | MD 3995.16 higher (3113.99 higher to 4876.34 higher) | ⨁⨁⨁◯ Moderate |  |

CI: confidence interval; RR: risk ratio

## Month Regimen- High Dose (200 ug)

| Certainty assessment | | | | | | | | | | | № of patients | | Effect | | Certainty | Importance |  |
| --- | --- | --- | --- | --- | --- | --- | --- | --- | --- | --- | --- | --- | --- | --- | --- | --- | --- |
| № of studies | | Study design | | Risk of bias | | Inconsistency | | Indirectness | Imprecision | Other considerations | Month Regimen 200 ug | [placebo] | Relative (95% CI) | Absolute (95% CI) |  |  |  |
| Pain | | | | | | | | | | | | | | | | |  |
| 5 | | randomised trials | | not serious | | not serious | | not serious | not serious | publication bias strongly suspected very strong association | 3894/9802 (39.7%) | 1050/9146 (11.5%) | **RR 8.18** (2.28 to 29.38) | **824 more per 1,000** (from 147 more to 1,000 more) | ⨁⨁⨁⨁ High |  |  |
| Swelling | | | | | | | | | | | | | | | | |  |
| 4 | | randomised trials | | not serious | | not serious | | not serious | not serious | strong association | 1177/9790 (12.0%) | 177/9124 (1.9%) | **RR 6.44** (5.52 to 7.52) | **106 more per 1,000** (from 88 more to 126 more) | ⨁⨁⨁⨁ High |  |  |
| Erythema | | | | | | | | | | | | | | | | |  |
| 4 | | randomised trials | | not serious | | not serious | | not serious | not serious | strong association | 904/9790 (9.2%) | 178/9124 (2.0%) | **RR 4.93** (4.21 to 5.78) | **77 more per 1,000** (from 63 more to 93 more) | ⨁⨁⨁⨁ High |  |  |
| Malaise/Fatigue | | | | | | | | | | | | | | | | |  |
| 5 | | randomised trials | | serious | | not serious | | not serious | serious | none | 3448/9802 (35.2%) | 2936/9146 (32.1%) | **RR 1.20** (0.94 to 1.53) | **64 more per 1,000** (from 19 fewer to 170 more) | ⨁⨁◯◯ Low |  |  |
| Myalgia | | | | | | | | | | | | | | | | |  |
| 5 | | randomised trials | | not serious | | not serious | | serious | not serious | none | 1912/9802 (19.5%) | 1517/9146 (16.6%) | **RR 1.10** (0.80 to 1.51) | **17 more per 1,000** (from 33 fewer to 85 more) | ⨁⨁⨁◯ Moderate |  |  |
| Headache | | | | | | | | | | | | | | | | |  |
| 4 | | randomised trials | | not serious | | not serious | | not serious | serious | none | 2758/9790 (28.2%) | 2386/9124 (26.2%) | **RR 1.24** (0.92 to 1.65) | **63 more per 1,000** (from 21 fewer to 170 more) | ⨁⨁⨁◯ Moderate |  |  |
| Arthralgia | | | | | | | | | | | | | | | | |  |
| 4 | | randomised trials | | not serious | | not serious | | not serious | serious | publication bias strongly suspected | 1639/9790 (16.7%) | 1416/9124 (15.5%) | **RR 1.16** (0.83 to 1.60) | **25 more per 1,000** (from 26 fewer to 93 more) | ⨁⨁◯◯ Low |  |  |
| Infections and Infestations | | | | | | | | | | | | | | | | |  |
| 2 | | randomised trials | | serious | | not serious | | not serious | not serious | none | 6/995 (0.6%) | 9/353 (2.5%) | RR 0.46 (0.14 to 1.52) | 14 fewer per 1,000 (from 22 fewer to 13 more) | ⨁⨁⨁◯ Moderate |  |  |
| Gastrointestinal Disorders | | | | | | | | | | | | | | | | |  |
| 4 | | randomised trials | | not serious | | not serious | | not serious | not serious | none | 193/9776 (2.0%) | 185/9129 (2.0%) | **RR 1.00** (0.82 to 1.23) | **0 fewer per 1,000** (from 4 fewer to 5 more) | ⨁⨁⨁⨁ High |  |  |
| Skin and Subcutaneous Tissue Disorders | | | | | | | | | | | | | | | | |  |
| 2 | | randomised trials | | serious | | not serious | | not serious | serious | none | 6/995 (0.6%) | 5/353 (1.4%) | RR 1.06 (0.05 to 21.66) | 1 more per 1,000 (from 13 fewer to 293 more) | ⨁⨁◯◯ Low |  |  |
| Respiratory, Thoracic, and Mediastinal Disorders | | | | | | | | | | | | | | | | |  |
| 2 | | randomised trials | | not serious | | not serious | | not serious | not serious | none | 80/995 (8.0%) | 30/353 (8.5%) | RR 1.01 (0.64 to 1.59) | 1 more per 1,000 (from 31 fewer to 50 more) | ⨁⨁⨁⨁ High |  |  |
| Bones/Joints/Musculoskeletal System-Related Problems | | | | | | | | | | | | | | | | |  |
| 2 | | randomised trials | | serious | | not serious | | not serious | not serious | strong association | 20/995 (2.0%) | 5/353 (1.4%) | RR 2.55 (0.96 to 6.81) | 22 more per 1,000 (from 1 fewer to 82 more) | ⨁⨁⨁⨁ High |  |  |
| Serious Adverse Events | | | | | | | | | | | | | | | | |  |
| 4 | | randomised trials | | serious | | not serious | | not serious | not serious | none | 1346/9788 (13.8%) | 1288/9123 (14.1%) | **RR 1.02** (0.95 to 1.09) | **3 more per 1,000** (from 7 fewer to 13 more) | ⨁⨁⨁◯ Moderate |  |  |
| Mortality | | | | | | | | | | | | | | | | |  |
| 4 | | randomised trials | | serious | | not serious | | not serious | serious | none | 214/9788 (2.2%) | 198/9123 (2.2%) | **RR 0.92** (0.42 to 2.02) | **2 fewer per 1,000** (from 13 fewer to 22 more) | ⨁⨁◯◯ Low |  |  |
| Adverse Events | | | | | | | | | | | | | | | | |  |
| 5 | | randomised trials | | serious | | not serious | | not serious | not serious | none | 4937/9800 (50.4%) | 4593/9145 (50.2%) | **RR 1.02** (0.99 to 1.05) | **10 more per 1,000** (from 5 fewer to 25 more) | ⨁⨁⨁◯ Moderate |  |  |
| Geometric Mean Concentration Anti Toxin - A (Neutralization units/ml) | | | | | | | | | | | | | | | | |  |
| 4 | | randomised trials | | not serious | | not serious | | not serious | serious | none | 1092 | 450 | - | MD 1246.69 higher (669.49 higher to 1823.89 higher) | ⨁⨁⨁◯ Moderate |  |  |
| Geometric Mean Concentration Anti Toxin - B (Neutralization units/ml) | | | | | | | | | | | | | | | | |  |
| 4 | | randomised trials | | not serious | | not serious | | not serious | serious | none | 1092 | 450 | - | MD 6700.23 higher (3974.83 higher to 9425.62 higher) | ⨁⨁⨁◯ Moderate |  |  |
| Mean percentage of the seroconverted population (Anti Toxin - A) | | | | | | | | | | | | | | | | |  |
| 2 | | randomised trials | | not serious | | not serious | | not serious | serious | none | 1044 | 390 | - | MD 66.59 higher (63.1 higher to 70.07 higher) | ⨁⨁⨁◯ Moderate |  |  |
| Mean percentage of the seroconverted population (Anti Toxin - B) | | | | | | | | | | | | | | | | |  |
| 2 | | randomised trials | | not serious | | not serious | | not serious | serious | none | 1044 | 390 | - | MD 77.97 higher (74.59 higher to 81.34 higher) | ⨁⨁⨁◯ Moderate |  |  |
| **Efficacy Analysis (Post Intervention Frequency of CDI)** | | | | | | | | | | | | | | | | | |
| 2 | | randomised trials | | not serious | | not serious | | not serious | | serious | none | 51/13897 (0.4%) | 41/10903 (0.4%) | **RR 0.86** (0.56 to 1.32) | **1 fewer per 1,000** (from 2 fewer to 1 more) | ⨁⨁⨁◯ Moderate |  |
| **Efficacy Analysis (Post Intervention Frequency of severe CDI)** | | | | | | | | | | | | | | | | | |
| 2 | | randomised trials | | not serious | | not serious | | not serious | | serious | none | 9/13897 (0.1%) | 17/10903 (0.2%) | **RR 0.25** (0.02 to 3.75) | **1 fewer per 1,000** (from 2 fewer to 4 more) | ⨁⨁⨁◯ Moderate |  |

CI: confidence interval; MD: mean difference; RR: risk ratio, CDI: *Clostridium Difficile* Infection
