# Supplementary material for: Comprehensive Evaluation of the Efficacy and Safety of the Clostridioides difficile Toxoid Vaccine: A Meta‐Analysis
Source: Can J Infect Dis Med Microbiol. 2026 Jul 30;2026:1160340. doi: 10.1155/cjid/1160340 (PMC13422635; doi:10.1155/cjid/1160340)
Supplement: Supplementary file 2 — Supporting Information 2 Supporting Figure 1. Forest plots for local adverse events (pain, swelling, erythema) in day‐regimen studies comparing Clostridioides difficile toxoid vaccine versus placebo groups. Effect estimates are expressed as relative risks (RRs) with 95% confidence intervals (CIs) using a random‐effects model. Heterogeneity is reported using the I‐squared statistic. [file CJID-2026-1160340-s001.pdf]

Analysis 1.1: Pain

Study or Subgroup	Vaccine Events	Vaccine Total	Placebo Events	Placebo Total	Weight	Risk Ratio IV, Random, 95% CI
Bruyn 2020	463	1199	79	586	86.4%	2.86 [2.31 , 3.56]
Greenberg 2012	20	36	3	12	3.9%	2.22 [0.80 , 6.18]
Inoue 2019	4	14	0	8	0.5%	5.40 [0.33 , 89.02]
Kitchin 2020	42	182	2	61	2.1%	7.04 [1.76 , 28.22]
Lawrence 2021	29	79	1	27	1.1%	9.91 [1.42 , 69.32]
Matsuoka 2018	46	68	5	34	6.0%	4.60 [2.01 , 10.51]
**Total (Wald[a])**		**1578**		**728**	**100.0%**	**3.02 [2.47 , 3.70]**
Total events:	604		90			

Test for overall effect: Z = 10.74 (P < 0.00001)

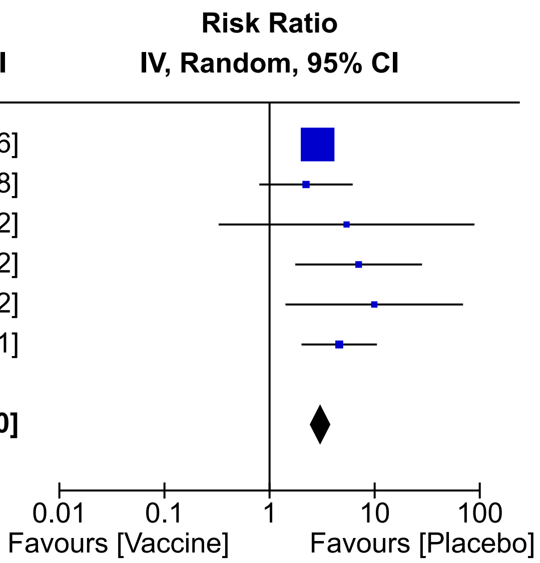

Heterogeneity: Tau (DL[b]) = 0.00; Chi = 4.60, df = 5 (P = 0.47); I = 0%

Footnotes

[a]CI calculated by Wald-type method.

[b]Tau calculated by DerSimonian and Laird method.

Analysis 1.2: Swelling

Study or Subgroup	Vaccine Events	Vaccine Total	Placebo Events	Placebo Total	Weight	Risk Ratio IV, Random, 95% CI
Bruyn 2020	45	1197	2	587	49.8%	11.03 [2.69 , 45.33]
Inoue 2019	5	14	0	8	12.9%	6.60 [0.41 , 105.81]
Kitchin 2020	5	182	0	61	12.0%	3.73 [0.21 , 66.44]
Lawrence 2021	12	79	0	27	12.7%	8.75 [0.54 , 142.99]
Matsuoka 2018	8	68	0	34	12.5%	8.62 [0.51 , 145.11]
**Total (Wald[a])**		**1540**		**717**	**100.0%**	**8.53 [3.15 , 23.14]**
Total events:	75		2			

Test for overall effect: Z = 4.21 (P 0.0001)

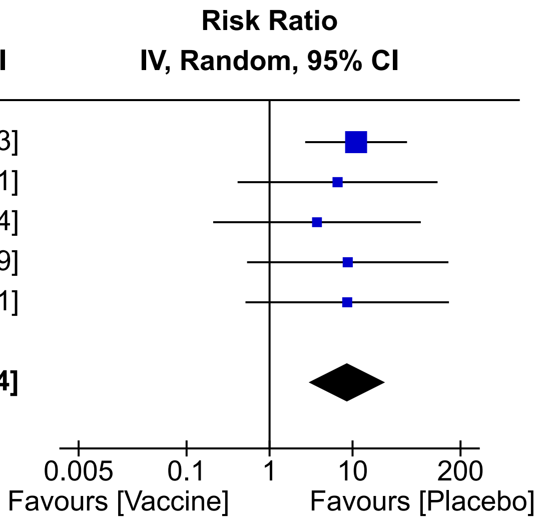

Heterogeneity: Tau (DL[b]) = 0.00; Chi = 0.48, df = 4 (P = 0.98); I = 0%

Footnotes

[a]CI calculated by Wald-type method.

[b]Tau calculated by DerSimonian and Laird method.

Analysis 1.3: Erythema

Study or Subgroup	Vaccine Events	Vaccine Total	Placebo Events	Placebo Total	Weight	Risk Ratio IV, Random, 95% CI
Bruyn 2020	50	1196	0	586	14.5%	49.53 [3.06 , 801.36]
Greenberg 2012	7	36	1	12	28.4%	2.33 [0.32 , 17.08]
Inoue 2019	8	14	0	8	15.1%	10.20 [0.67 , 156.37]
Kitchin 2020	4	182	0	61	13.3%	3.05 [0.17 , 55.83]
Lawrence 2021	8	79	0	27	14.2%	5.95 [0.35 , 99.77]
Matsuoka 2018	11	68	0	34	14.4%	11.67 [0.71 , 192.25]
**Total (Wald[a])**		**1575**		**728**	**100.0%**	**6.78 [2.35 , 19.61]**
Total events:	88		1			

Test for overall effect: Z = 3.53 (P = 0.0004)

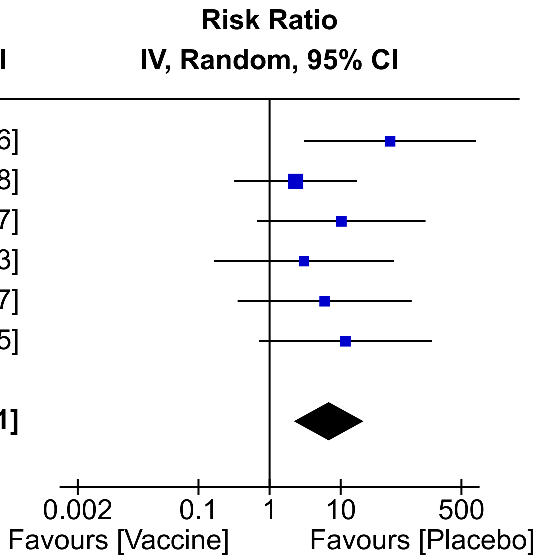

Heterogeneity: Tau (DL[b]) = 0.00; Chi = 3.59, df = 5 (P = 0.61); I = 0%

Footnotes

[a]CI calculated by Wald-type method.

[b]Tau calculated by DerSimonian and Laird method.
