# Supplementary material for: Comprehensive Evaluation of the Efficacy and Safety of the Clostridioides difficile Toxoid Vaccine: A Meta‐Analysis
Source: Can J Infect Dis Med Microbiol. 2026 Jul 30;2026:1160340. doi: 10.1155/cjid/1160340 (PMC13422635; doi:10.1155/cjid/1160340)
Supplement: Supplementary file 3 — Supporting Information 3 Supporting Figure 2. Forest plots for systemic adverse events (fever, fatigue/malaise, myalgia, headache, arthralgia) in day‐regimen studies comparing vaccine versus placebo groups. Effect estimates are expressed as RR with 95% CI using a random‐effects model. [file CJID-2026-1160340-s002.pdf]

Analysis 2.1: Fever

Study or Subgroup	Vaccine Events	Vaccine Total	Placebo Events	Placebo Total	Weight	Risk Ratio IV, Random, 95% CI
Bruyn 2020	56	1173	33	577	98.1%	0.83 [0.55 , 1.27]
Matsuoka 2018	2	68	0	34	1.9%	2.54 [0.13 , 51.40]
**Total (Wald[a])**		**1241**		**611**	**100.0%**	**0.85 [0.56 , 1.29]**
Total events:	58		33			

Test for overall effect: Z = 0.75 (P = 0.45)

Heterogeneity: Tau (DL[b]) = 0.00; Chi = 0.51, df = 1 (P = 0.47); I = 0%

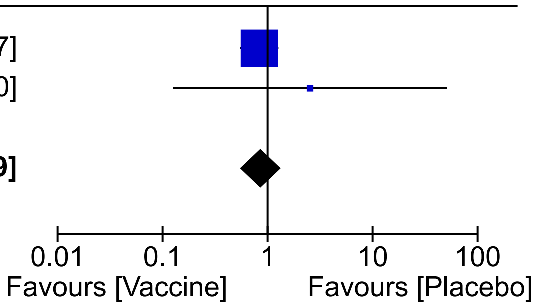

Footnotes

[a]CI calculated by Wald-type method.

[b]Tau calculated by DerSimonian and Laird method.

Analysis 2.2: Malaise/Fatigue

Study or Subgroup	Vaccine Events	Vaccine Total	Placebo Events	Placebo Total	Weight	Risk Ratio IV, Random, 95% CI
Bruyn 2020	286	1196	115	586	46.1%	1.22 [1.01 , 1.48]
Greenberg 2012	7	36	2	12	8.2%	1.17 [0.28 , 4.87]
Kitchin 2020	18	182	11	61	22.8%	0.55 [0.27 , 1.10]
Lawrence 2021	26	79	4	27	15.2%	2.22 [0.85 , 5.79]
Matsuoka 2018	8	68	2	34	7.6%	2.00 [0.45 , 8.91]
**Total (Wald[a])**		**1561**		**720**	**100.0%**	**1.15 [0.74 , 1.80]**
Total events:	345		134			

Test for overall effect: Z = 0.62 (P = 0.54)

Heterogeneity: Tau (DL[b]) = 0.10; Chi = 6.97, df = 4 (P = 0.14); I = 43%

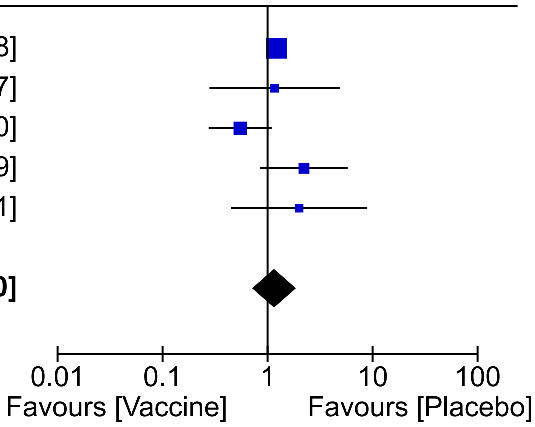

Footnotes

[a]CI calculated by Wald-type method.

[b]Tau calculated by DerSimonian and Laird method.

Analysis 2.3: Myalgia

Study or Subgroup	Vaccine Events	Vaccine Total	Placebo Events	Placebo Total	Weight	Risk Ratio IV, Random, 95% CI
Bruyn 2020	318	1196	124	586	92.8%	1.26 [1.05 , 1.51]
Greenberg 2012	5	36	2	12	1.4%	0.83 [0.19 , 3.75]
Inoue 2019	1	14	0	8	0.3%	1.80 [0.08 , 39.64]
Kitchin 2020	9	182	2	61	1.4%	1.51 [0.34 , 6.79]
Lawrence 2021	15	79	3	27	2.3%	1.71 [0.54 , 5.45]
Matsuoka 2018	7	68	3	34	1.9%	1.17 [0.32 , 4.23]
**Total (Wald[a])**		**1575**		**728**	**100.0%**	**1.26 [1.06 , 1.50]**
Total events:	355		134			

Test for overall effect: Z = 2.59 (P = 0.010)

Heterogeneity: Tau (DL[b]) = 0.00; Chi = 0.68, df = 5 (P = 0.98); I = 0%

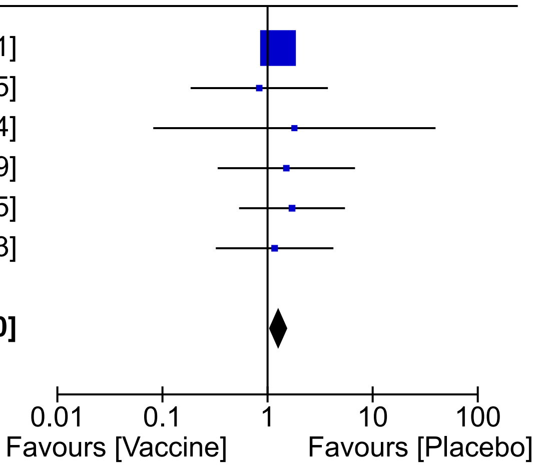

Footnotes

[a]CI calculated by Wald-type method.

[b]Tau calculated by DerSimonian and Laird method.
