# Supplementary material for: Comprehensive Evaluation of the Efficacy and Safety of the Clostridioides difficile Toxoid Vaccine: A Meta‐Analysis
Source: Can J Infect Dis Med Microbiol. 2026 Jul 30;2026:1160340. doi: 10.1155/cjid/1160340 (PMC13422635; doi:10.1155/cjid/1160340)
Supplement: Supplementary file 4 — Supporting Information 4 Supporting Figure 3. Forest plots for overall adverse events, serious adverse events, and mortality in day‐regimen studies comparing vaccine versus placebo groups. Effect estimates are expressed as RR with 95% CI using a random‐effects model. [file CJID-2026-1160340-s003.pdf]

Analysis 2.4: Headache

Study or Subgroup	Vaccine Events	Vaccine Total	Placebo Events	Placebo Total	Weight	Risk Ratio IV, Random, 95% CI
Bruyn 2020	302	1196	132	586	92.4%	1.12 [0.94 , 1.34]
Kitchin 2020	15	182	7	61	4.1%	0.72 [0.31 , 1.68]
Lawrence 2021	14	79	4	27	2.8%	1.20 [0.43 , 3.32]
Matsuoka 2018	6	68	1	34	0.7%	3.00 [0.38 , 23.93]
**Total (Wald[a])**		**1525**		**708**	**100.0%**	**1.11 [0.93 , 1.32]**
Total events:	337		144			

Risk Ratio IV, Random, 95% CI

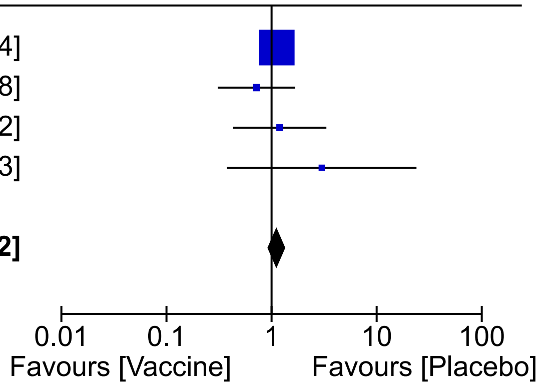

Test for overall effect: Z = 1.19 (P = 0.23)

Heterogeneity: Tau (DL[b]) = 0.00; Chi = 1.92, df = 3 (P = 0.59); I = 0%

Footnotes

[a]CI calculated by Wald-type method.

[b]Tau calculated by DerSimonian and Laird method.

Analysis 2.5: Arthralgia

Study or Subgroup	Vaccine Events	Vaccine Total	Placebo Events	Placebo Total	Weight	Risk Ratio IV, Random, 95% CI
Bruyn 2020	233	1196	98	586	94.4%	1.16 [0.94 , 1.44]
Greenberg 2012	3	36	2	12	1.6%	0.50 [0.09 , 2.64]
Kitchin 2020	7	182	3	61	2.5%	0.78 [0.21 , 2.93]
Lawrence 2021	16	79	1	27	1.1%	5.47 [0.76 , 39.31]
Matsuoka 2018	2	68	0	34	0.5%	2.54 [0.13 , 51.40]
**Total (Wald[a])**		**1561**		**720**	**100.0%**	**1.16 [0.94 , 1.43]**
Total events:	261		104			

Risk Ratio IV, Random, 95% CI

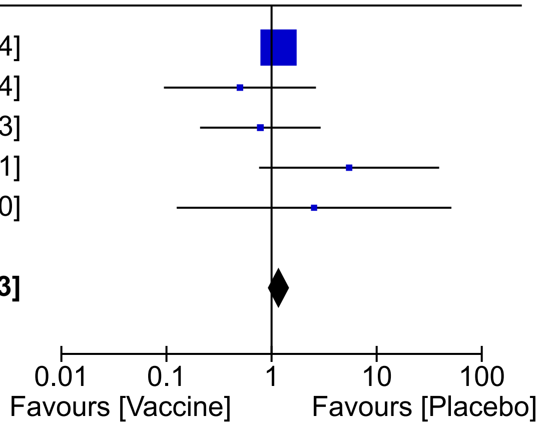

Test for overall effect: Z = 1.42 (P = 0.16)

Heterogeneity: Tau (DL[b]) = 0.00; Chi = 3.96, df = 4 (P = 0.41); I = 0%

Footnotes

[a]CI calculated by Wald-type method.

[b]Tau calculated by DerSimonian and Laird method.

Analysis 3.1: Infections and Infestations

Study or Subgroup	Vaccine Events	Vaccine Total	Placebo Events	Placebo Total	Weight	Risk Ratio IV, Random, 95% CI
Bruyn 2020	892	6113	419	3057	99.8%	1.06 [0.96 , 1.19]
Matsuoka 2018	1	68	1	34	0.2%	0.50 [0.03 , 7.75]
**Total (Wald[a])**		**6181**		**3091**	**100.0%**	**1.06 [0.95 , 1.18]**
Total events:	893		420			

Risk Ratio IV, Random, 95% CI

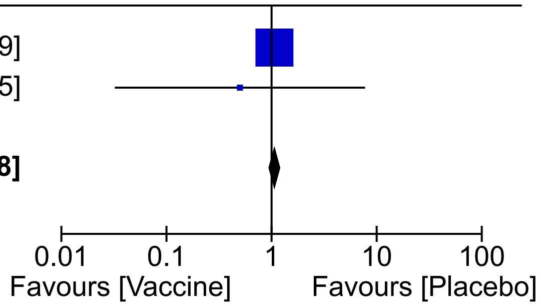

Test for overall effect: Z = 1.12 (P = 0.26)

Heterogeneity: Tau (DL[b]) = 0.00; Chi = 0.29, df = 1 (P = 0.59); I = 0%

Footnotes

[a]CI calculated by Wald-type method.

[b]Tau calculated by DerSimonian and Laird method.
