# Supplementary material for: Comprehensive Evaluation of the Efficacy and Safety of the Clostridioides difficile Toxoid Vaccine: A Meta‐Analysis
Source: Can J Infect Dis Med Microbiol. 2026 Jul 30;2026:1160340. doi: 10.1155/cjid/1160340 (PMC13422635; doi:10.1155/cjid/1160340)
Supplement: Supplementary file 5 — Supporting Information 5 Supporting Figure 4. Forest plots for infections/infestations, gastrointestinal disorders, musculoskeletal complaints, and respiratory disorders in day‐regimen studies. Effect estimates are expressed as RR with 95% CI using a random‐effects model. [file CJID-2026-1160340-s004.pdf]

Analysis 3.2: GI

Study or Subgroup	Vaccine Events	Vaccine Total	Placebo Events	Placebo Total	Weight	Risk Ratio IV, Random, 95% CI
Bruyn 2020	699	6113	375	3057	39.6%	0.93 [0.83 , 1.05]
Greenberg 2012	1	36	2	12	9.1%	0.17 [0.02 , 1.68]
Kitchin 2020	7	182	10	61	25.9%	0.23 [0.09 , 0.59]
Lawrence 2021	12	79	5	27	25.4%	0.82 [0.32 , 2.12]
**Total (Wald[a])**		**6410**		**3157**	**100.0%**	**0.54 [0.24 , 1.19]**
Total events:	719		392			

Test for overall effect: Z = 1.53 (P = 0.13)

Heterogeneity: Tau (DL[b]) = 0.41; Chi = 10.59, df = 3 (P = 0.01); I = 72%

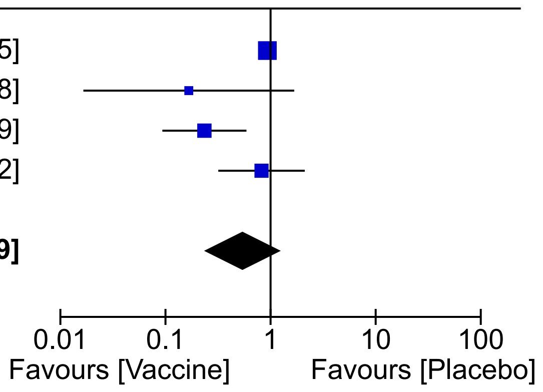

Footnotes

[a]CI calculated by Wald-type method.
[b]Tau calculated by DerSimonian and Laird method.

Analysis 3.3: MSK

Study or Subgroup	Vaccine Events	Vaccine Total	Placebo Events	Placebo Total	Weight	Risk Ratio IV, Random, 95% CI
Bruyn 2020	425	6113	203	3057	99.4%	1.05 [0.89 , 1.23]
Greenberg 2012	2	36	0	12	0.3%	1.76 [0.09 , 34.24]
Matsuoka 2018	2	68	0	34	0.3%	2.54 [0.13 , 51.40]
**Total (Wald[a])**		**6217**		**3103**	**100.0%**	**1.05 [0.89 , 1.23]**
Total events:	429		203			

Test for overall effect: Z = 0.61 (P = 0.54)

Heterogeneity: Tau (DL[b]) = 0.00; Chi = 0.45, df = 2 (P = 0.80); I = 0%

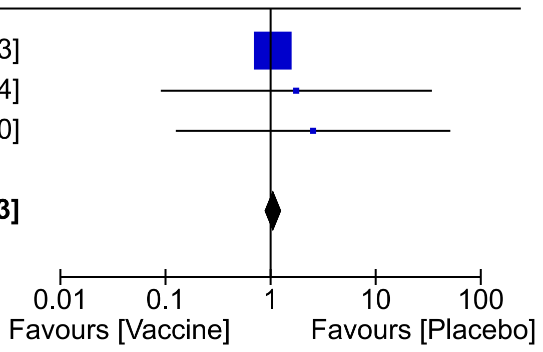

Footnotes

[a]CI calculated by Wald-type method.
[b]Tau calculated by DerSimonian and Laird method.

Analysis 3.4: Injury and Poisoning

Study or Subgroup	Vaccine Events	Vaccine Total	Placebo Events	Placebo Total	Weight	Risk Ratio IV, Random, 95% CI
Bruyn 2020	309	6113	138	3057	99.6%	1.12 [0.92 , 1.36]
Matsuoka 2018	1	68	0	34	0.4%	1.52 [0.06 , 36.40]
**Total (Wald[a])**		**6181**		**3091**	**100.0%**	**1.12 [0.92 , 1.36]**
Total events:	310		138			

Test for overall effect: Z = 1.15 (P = 0.25)

Heterogeneity: Tau (DL[b]) = 0.00; Chi = 0.04, df = 1 (P = 0.85); I = 0%

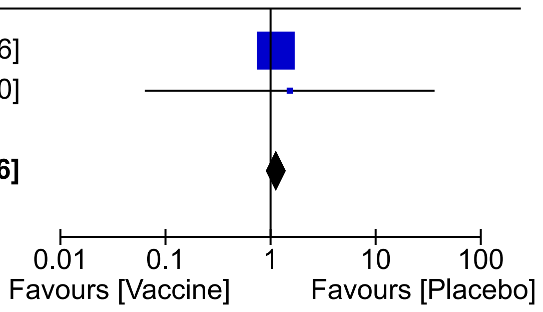

Footnotes

[a]CI calculated by Wald-type method.
[b]Tau calculated by DerSimonian and Laird method.

Analysis 3.5: Serious Adverse Events

Study or Subgroup	Vaccine Events	Vaccine Total	Placebo Events	Placebo Total	Weight	Risk Ratio IV, Random, 95% CI
Bruyn 2020	1662	6113	851	3057	99.9%	0.98 [0.91 , 1.05]
Lawrence 2021	2	18	1	6	0.1%	0.67 [0.07 , 6.11]
**Total (Wald[a])**		**6131**		**3063**	**100.0%**	**0.98 [0.91 , 1.05]**
Total events:	1664		852			

Test for overall effect: Z = 0.67 (P = 0.50)

Heterogeneity: Tau (DL[b]) = 0.00; Chi = 0.11, df = 1 (P = 0.74); I = 0%

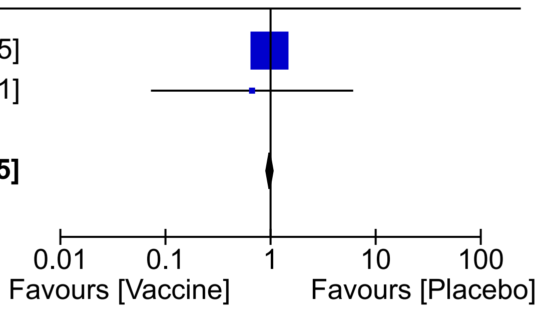

Footnotes

[a]CI calculated by Wald-type method.
[b]Tau calculated by DerSimonian and Laird method.

Analysis 3.6: Adverse Events

Study or Subgroup	Vaccine Events	Vaccine Total	Placebo Events	Placebo Total	Weight	Risk Ratio IV, Random, 95% CI
Inoue 2019	7	14	0	8	29.6%	9.00 [0.58 , 139.50]
Lawrence 2021	28	79	9	27	70.4%	1.06 [0.58 , 1.96]
**Total (Wald[a])**		**93**		**35**	**100.0%**	**2.00 [0.30 , 13.54]**
Total events:	35		9			

Test for overall effect: Z = 0.71 (P = 0.48)

Heterogeneity: Tau (DL[b]) = 1.25; Chi = 2.22, df = 1 (P = 0.14); I = 55%

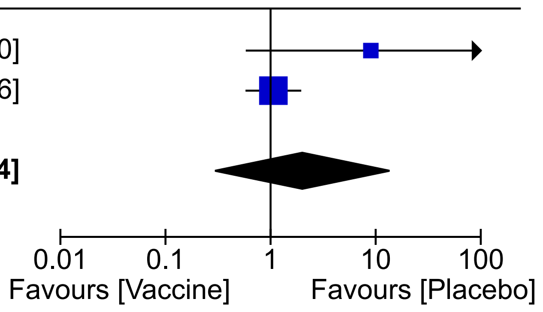

Footnotes

[a]CI calculated by Wald-type method.
[b]Tau calculated by DerSimonian and Laird method.
