# Supplementary material for: Comprehensive Evaluation of the Efficacy and Safety of the Clostridioides difficile Toxoid Vaccine: A Meta‐Analysis
Source: Can J Infect Dis Med Microbiol. 2026 Jul 30;2026:1160340. doi: 10.1155/cjid/1160340 (PMC13422635; doi:10.1155/cjid/1160340)
Supplement: Supplementary file 6 — Supporting Information 6 Supporting Figure 5. Forest plots for local adverse events (pain, swelling, erythema) in month‐regimen studies receiving 100‐μg vaccine doses versus placebo. Effect estimates are expressed as RR with 95% CI using a random‐effects model. [file CJID-2026-1160340-s013.pdf]

Analysis 1.1: Pain

Study or Subgroup	Vaccine Events	Vaccine Total	Placebo Events	Placebo Total	Weight	Risk Ratio IV, Random, 95% CI
Christensen 2023	228	983	14	331	72.5%	5.48 [3.24 , 9.27]
Inoue 2019	0	24	0	16		Not estimable
Kitchin 2020	40	183	1	61	19.1%	13.33 [1.87 , 94.95]
Sheldon 2016	0	24	1	44	8.4%	0.60 [0.03 , 14.19]
**Total (Wald[a])**		**1214**		**452**	**100.0%**	**5.39 [2.06 , 14.11]**
Total events:	268		16			

Test for overall effect: Z = 3.43 (P = 0.0006)

Heterogeneity: Tau (DL[b]) = 0.26; Chi = 2.67, df = 2 (P = 0.26); I = 25%

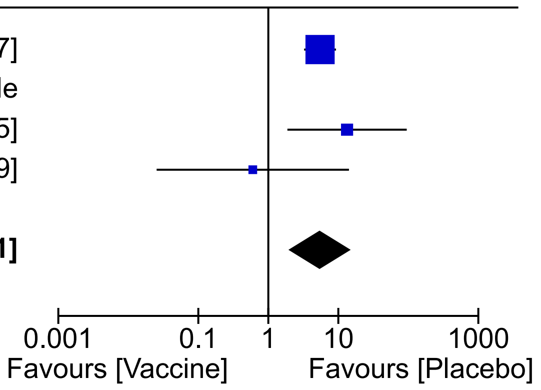

Footnotes
[a]CI calculated by Wald-type method.
[b]Tau calculated by DerSimonian and Laird method.

Analysis 1.2: Swelling

Study or Subgroup	Vaccine Events	Vaccine Total	Placebo Events	Placebo Total	Weight	Risk Ratio IV, Random, 95% CI
Christensen 2023	46	983	2	331	61.0%	7.74 [1.89 , 31.73]
Inoue 2019	0	24	0	16		Not estimable
Kitchin 2020	5	183	1	61	26.8%	1.67 [0.20 , 13.99]
Sheldon 2016	1	24	0	44	12.1%	5.40 [0.23 , 127.67]
**Total (Wald[a])**		**1214**		**452**	**100.0%**	**4.91 [1.63 , 14.78]**
Total events:	52		3			

Test for overall effect: Z = 2.83 (P = 0.005)

Heterogeneity: Tau (DL[b]) = 0.00; Chi = 1.40, df = 2 (P = 0.50); I = 0%

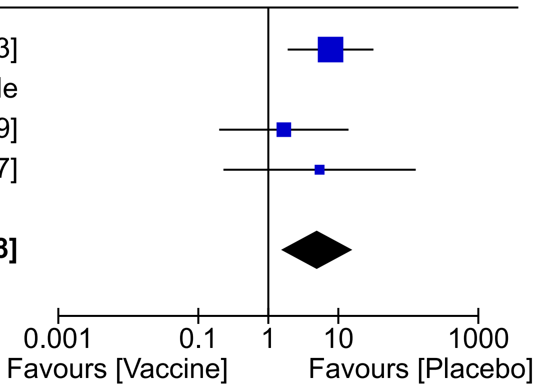

Footnotes
[a]CI calculated by Wald-type method.
[b]Tau calculated by DerSimonian and Laird method.

Analysis 1.3: Erythema

Study or Subgroup	Vaccine Events	Vaccine Total	Placebo Events	Placebo Total	Weight	Risk Ratio IV, Random, 95% CI
Christensen 2023	36	983	3	331	85.5%	4.04 [1.25 , 13.03]
Inoue 2019	0	24	0	16		Not estimable
Kitchin 2020	7	183	0	61	14.5%	5.05 [0.29 , 87.22]
Sheldon 2016	0	24	0	44		Not estimable
**Total (Wald[a])**		**1214**		**452**	**100.0%**	**4.17 [1.41 , 12.33]**
Total events:	43		3			

Test for overall effect: Z = 2.59 (P = 0.010)

Heterogeneity: Tau (DL[b]) = 0.00; Chi = 0.02, df = 1 (P = 0.89); I = 0%

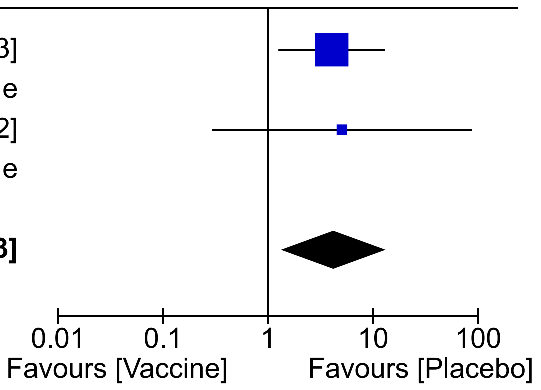

Footnotes
[a]CI calculated by Wald-type method.
[b]Tau calculated by DerSimonian and Laird method.

Analysis 2.1: Malaise/Fatigue

Study or Subgroup	Vaccine Events	Vaccine Total	Placebo Events	Placebo Total	Weight	Risk Ratio IV, Random, 95% CI
Christensen 2023	5	983	4	331	29.7%	0.42 [0.11 , 1.56]
Kitchin 2020	38	183	9	61	63.6%	1.41 [0.72 , 2.74]
Sheldon 2016	0	24	1	44	6.6%	0.60 [0.03 , 14.19]
**Total (Wald[a])**		**1190**		**436**	**100.0%**	**0.93 [0.40 , 2.16]**
Total events:	43		14			

Test for overall effect: Z = 0.17 (P = 0.86)

Heterogeneity: Tau (DL[b]) = 0.17; Chi = 2.73, df = 2 (P = 0.26); I = 27%

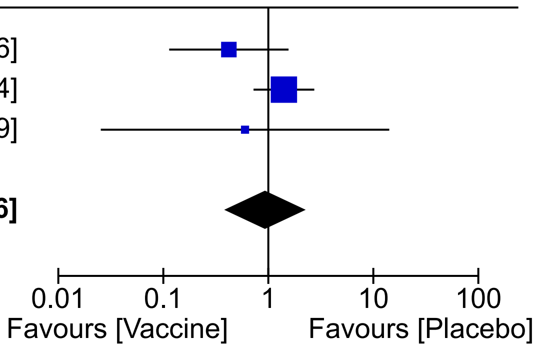

Footnotes
[a]CI calculated by Wald-type method.
[b]Tau calculated by DerSimonian and Laird method.
