# Supplementary material for: Comprehensive Evaluation of the Efficacy and Safety of the Clostridioides difficile Toxoid Vaccine: A Meta‐Analysis
Source: Can J Infect Dis Med Microbiol. 2026 Jul 30;2026:1160340. doi: 10.1155/cjid/1160340 (PMC13422635; doi:10.1155/cjid/1160340)
Supplement: Supplementary file 7 — Supporting Information 7 Supporting Figure 6. Forest plots for systemic adverse events (fatigue, myalgia, headache, malaise, arthralgia) in month‐regimen studies receiving 100‐μg vaccine doses. Effect estimates are expressed as RR with 95% CI using a random‐effects model. [file CJID-2026-1160340-s006.pdf]

Analysis 2.2: Myalgia

Study or Subgroup	Vaccine Events	Vaccine Total	Placebo Events	Placebo Total	Weight	Risk Ratio IV, Random, 95% CI
Christensen 2023	76	983	19	331	73.9%	1.35 [0.83 , 2.19]
Kitchin 2020	15	183	7	61	24.3%	0.71 [0.31 , 1.67]
Sheldon 2016	0	24	1	44	1.8%	0.60 [0.03 , 14.19]
**Total (Wald[a])**		**1190**		**436**	**100.0%**	**1.14 [0.75 , 1.73]**
Total events:	91		27			

Test for overall effect: Z = 0.61 (P = 0.54)

Heterogeneity: Tau (DL[b]) = 0.00; Chi = 1.77, df = 2 (P = 0.41); I = 0%

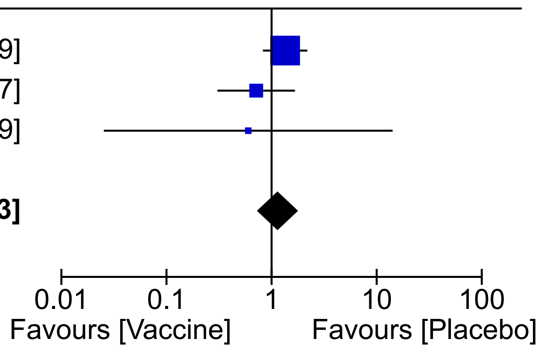

Footnotes

[a]CI calculated by Wald-type method.

[b]Tau calculated by DerSimonian and Laird method.

Analysis 2.3: Arthralgia

Study or Subgroup	Vaccine Events	Vaccine Total	Placebo Events	Placebo Total	Weight	Risk Ratio IV, Random, 95% CI
Christensen 2023	13	983	4	331	34.6%	1.09 [0.36 , 3.33]
Kitchin 2020	20	183	7	61	65.4%	0.95 [0.42 , 2.14]
**Total (Wald[a])**		**1166**		**392**	**100.0%**	**1.00 [0.52 , 1.92]**
Total events:	33		11			

Test for overall effect: Z = 0.00 (P = 1.00)

Heterogeneity: Tau (DL[b]) = 0.00; Chi = 0.04, df = 1 (P = 0.84); I = 0%

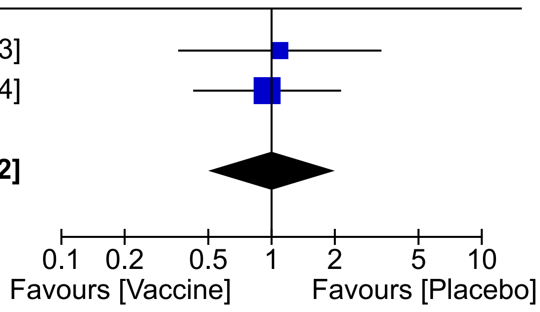

Footnotes

[a]CI calculated by Wald-type method.

[b]Tau calculated by DerSimonian and Laird method.

Analysis 2.4: Headache

Study or Subgroup	Vaccine Events	Vaccine Total	Placebo Events	Placebo Total	Weight	Risk Ratio IV, Random, 95% CI
Christensen 2023	135	983	4	331	49.1%	11.36 [4.24 , 30.48]
Kitchin 2020	24	183	11	61	50.9%	0.73 [0.38 , 1.40]
**Total (Wald[a])**		**1166**		**392**	**100.0%**	**2.80 [0.19 , 41.41]**
Total events:	159		15			

Test for overall effect: Z = 0.75 (P = 0.45)

Heterogeneity: Tau (DL[b]) = 3.60; Chi = 20.76, df = 1 (P 0.00001); I = 95%

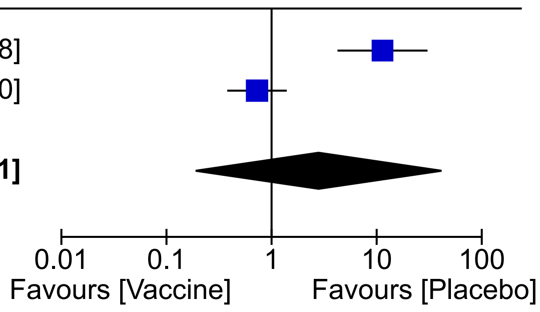

Footnotes

[a]CI calculated by Wald-type method.

[b]Tau calculated by DerSimonian and Laird method.

Analysis 3.1: Infections and Infestations

Study or Subgroup	Vaccine Events	Vaccine Total	Placebo Events	Placebo Total	Weight	Risk Ratio IV, Random, 95% CI
Christensen 2023	4	983	5	331	46.5%	0.27 [0.07 , 1.00]
Sheldon 2016	8	24	8	44	53.5%	1.83 [0.79 , 4.27]
**Total (Wald[a])**		**1007**		**375**	**100.0%**	**0.75 [0.12 , 4.90]**
Total events:	12		13			

Test for overall effect: Z = 0.30 (P = 0.77)

Heterogeneity: Tau (DL[b]) = 1.52; Chi = 5.82, df = 1 (P = 0.02); I = 83%

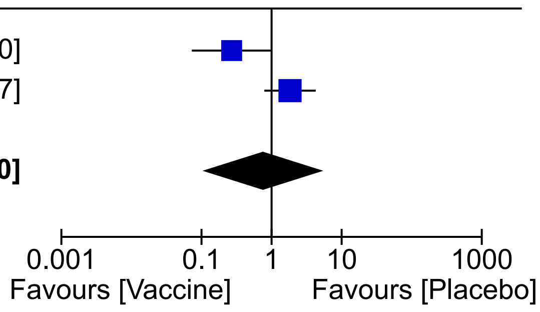

Footnotes

[a]CI calculated by Wald-type method.

[b]Tau calculated by DerSimonian and Laird method.

Analysis 3.2: GI

Study or Subgroup	Vaccine Events	Vaccine Total	Placebo Events	Placebo Total	Weight	Risk Ratio IV, Random, 95% CI
Christensen 2023	9	983	2	331	18.6%	1.52 [0.33 , 6.98]
Kitchin 2020	31	183	7	61	73.6%	1.48 [0.69 , 3.18]
Sheldon 2016	1	24	2	44	7.9%	0.92 [0.09 , 9.60]
**Total (Wald[a])**		**1190**		**436**	**100.0%**	**1.43 [0.74 , 2.76]**
Total events:	41		11			

Test for overall effect: Z = 1.06 (P = 0.29)

Heterogeneity: Tau (DL[b]) = 0.00; Chi = 0.15, df = 2 (P = 0.93); I = 0%

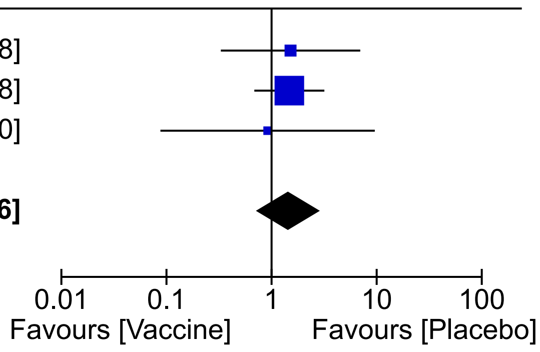

Footnotes

[a]CI calculated by Wald-type method.

[b]Tau calculated by DerSimonian and Laird method.
