# Supplementary material for: Comprehensive Evaluation of the Efficacy and Safety of the Clostridioides difficile Toxoid Vaccine: A Meta‐Analysis
Source: Can J Infect Dis Med Microbiol. 2026 Jul 30;2026:1160340. doi: 10.1155/cjid/1160340 (PMC13422635; doi:10.1155/cjid/1160340)
Supplement: Supplementary file 8 — Supporting Information 8 Supporting Figure 7. Forest plots for overall adverse events and serious adverse events in month‐regimen studies receiving 100‐μg vaccine doses. Effect estimates are expressed as RR with 95% CI using a random‐effects model. [file CJID-2026-1160340-s007.pdf]

Analysis 3.3: Skin

Study or Subgroup	Vaccine Events	Vaccine Total	Placebo Events	Placebo Total	Weight	Risk Ratio IV, Random, 95% CI
Christensen 2023	3	983	4	331	53.8%	0.25 [0.06 , 1.12]
Sheldon 2016	2	24	3	44	46.2%	1.22 [0.22 , 6.82]
**Total (Wald[a])**		**1007**		**375**	**100.0%**	**0.52 [0.11 , 2.44]**
Total events:	5		7			
Test for overall effect: Z = 0.82 (P = 0.41)						

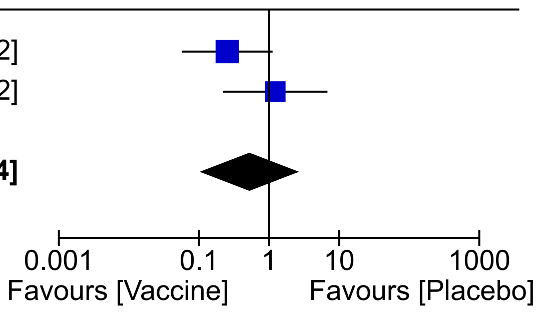

Heterogeneity: Tau (DL[b]) = 0.57; Chi = 1.84, df = 1 (P = 0.17); I = 46%

Footnotes

[a]CI calculated by Wald-type method.

[b]Tau calculated by DerSimonian and Laird method.

Analysis 3.4: Respiratory

Study or Subgroup	Vaccine Events	Vaccine Total	Placebo Events	Placebo Total	Weight	Risk Ratio IV, Random, 95% CI
Christensen 2023	40	983	13	331	88.7%	1.04 [0.56 , 1.91]
Sheldon 2016	2	24	3	44	11.3%	1.22 [0.22 , 6.82]
**Total (Wald[a])**		**1007**		**375**	**100.0%**	**1.06 [0.59 , 1.88]**
Total events:	42		16			
Test for overall effect: Z = 0.18 (P = 0.85)						

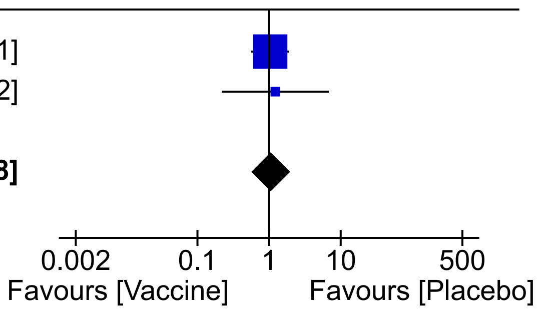

Heterogeneity: Tau (DL[b]) = 0.00; Chi = 0.03, df = 1 (P = 0.86); I = 0%

Footnotes

[a]CI calculated by Wald-type method.

[b]Tau calculated by DerSimonian and Laird method.

Analysis 3.5: MSK

Study or Subgroup	Vaccine Events	Vaccine Total	Placebo Events	Placebo Total	Weight	Risk Ratio IV, Random, 95% CI
Christensen 2023	11	983	4	331	77.1%	0.93 [0.30 , 2.89]
Sheldon 2016	1	24	5	44	22.9%	0.37 [0.05 , 2.96]
**Total (Wald[a])**		**1007**		**375**	**100.0%**	**0.75 [0.28 , 2.03]**
Total events:	12		9			
Test for overall effect: Z = 0.57 (P = 0.57)						

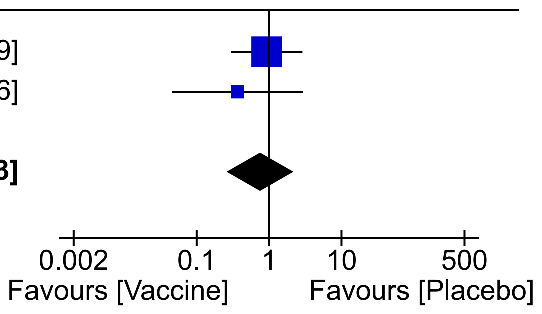

Heterogeneity: Tau (DL[b]) = 0.00; Chi = 0.58, df = 1 (P = 0.45); I = 0%

Footnotes

[a]CI calculated by Wald-type method.

[b]Tau calculated by DerSimonian and Laird method.

Analysis 3.6: Injury and Poisoning

Study or Subgroup	Vaccine Events	Vaccine Total	Placebo Events	Placebo Total	Weight	Risk Ratio IV, Random, 95% CI
Christensen 2023	12	983	2	331	68.7%	2.02 [0.45 , 8.98]
Sheldon 2016	3	24	1	44	31.3%	5.50 [0.60 , 50.03]
**Total (Wald[a])**		**1007**		**375**	**100.0%**	**2.77 [0.80 , 9.52]**
Total events:	15		3			
Test for overall effect: Z = 1.61 (P = 0.11)						

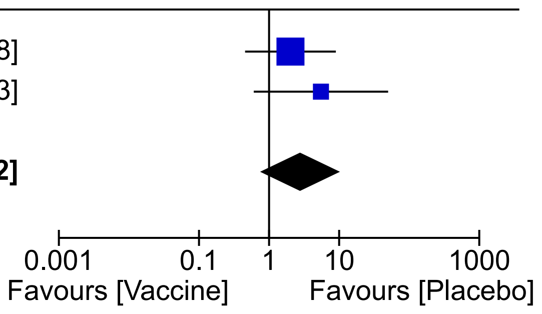

Heterogeneity: Tau (DL[b]) = 0.00; Chi = 0.54, df = 1 (P = 0.46); I = 0%

Footnotes

[a]CI calculated by Wald-type method.

[b]Tau calculated by DerSimonian and Laird method.

Analysis 3.7: Adverse Events

Study or Subgroup	Vaccine Events	Vaccine Total	Placebo Events	Placebo Total	Weight	Risk Ratio IV, Random, 95% CI
Christensen 2023	359	983	117	331	98.5%	1.03 [0.87 , 1.22]
Inoue 2019	4	24	3	16	1.5%	0.89 [0.23 , 3.45]
**Total (Wald[a])**		**1007**		**347**	**100.0%**	**1.03 [0.87 , 1.22]**
Total events:	363		120			
Test for overall effect: Z = 0.36 (P = 0.72)						

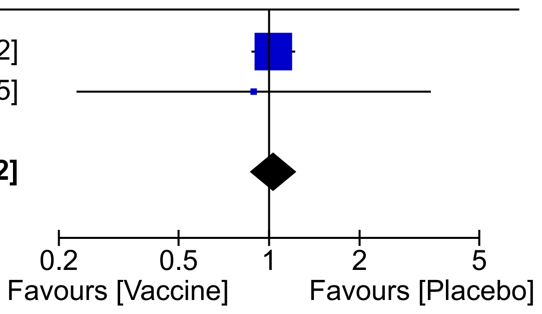

Heterogeneity: Tau (DL[b]) = 0.00; Chi = 0.05, df = 1 (P = 0.83); I = 0%

Footnotes

[a]CI calculated by Wald-type method.

[b]Tau calculated by DerSimonian and Laird method.

Analysis 3.8: Mortality

Study or Subgroup	Vaccine Events	Vaccine Total	Placebo Events	Placebo Total	Weight	Risk Ratio M-H, Random, 95% CI
Christensen 2023	2	983	2	331	100.0%	0.34 [0.05 , 2.38]
Inoue 2019	0	24	0	16		Not estimable
**Total**		**1007**		**347**	**100.0%**	**0.34 [0.05 , 2.38]**
Total events:	2		2			
Test for overall effect: Z = 1.09 (P = 0.28)						

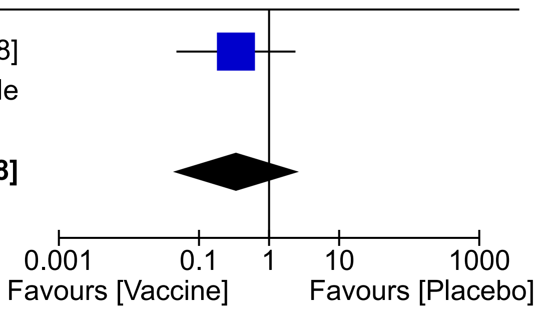

Heterogeneity: Not applicable
