# Supplementary material for: Comprehensive Evaluation of the Efficacy and Safety of the Clostridioides difficile Toxoid Vaccine: A Meta‐Analysis
Source: Can J Infect Dis Med Microbiol. 2026 Jul 30;2026:1160340. doi: 10.1155/cjid/1160340 (PMC13422635; doi:10.1155/cjid/1160340)
Supplement: Supplementary file 9 — Supporting Information 9 Supporting Figure 8. Forest plots for local adverse events (pain, swelling, erythema) in month‐regimen studies receiving 200‐μg vaccine doses versus placebo. Effect estimates are expressed as RR with 95% CI using a random‐effects model. [file CJID-2026-1160340-s008.pdf]

Analysis 5.1: Pain

Study or Subgroup	Clostridium Difficile Vaccine Events	Clostridium Difficile Vaccine Total	Normal Saline Placebo Events	Normal Saline Placebo Total	Weight	Risk Ratio IV, Random, 95% CI
Christensen 2023	213	985	3	332	29.2%	23.93 [7.71 , 74.28]
Donskey 2024	3663	8722	1046	8718	37.9%	3.50 [3.29 , 3.72]
Inoue 2019	6	24	1	16	19.5%	4.00 [0.53 , 30.16]
Kitchin 2020	12	59	0	58	13.4%	24.58 [1.49 , 405.79]
Sheldon 2016	0	12	0	22		Not estimable
**Total (Wald[a])**		**9802**		**9146**	**100.0%**	**8.18 [2.28 , 29.38]**
Total events:	3894		1050			

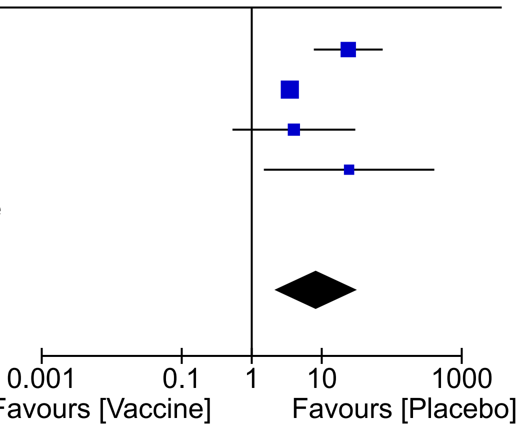

Test for overall effect: Z = 3.22 (P = 0.001)

Heterogeneity: Tau (DL[b]) = 1.12; Chi = 12.89, df = 3 (P = 0.005); I = 77%

Footnotes

[a]CI calculated by Wald-type method.

[b]Tau calculated by DerSimonian and Laird method.

Analysis 5.2: Swelling

Study or Subgroup	Clostridium Difficile Vaccine Events	Clostridium Difficile Vaccine Total	Normal Saline Placebo Events	Normal Saline Placebo Total	Weight	Risk Ratio IV, Random, 95% CI
Christensen 2023	41	985	3	332	1.8%	4.61 [1.44 , 14.78]
Donskey 2024	1134	8722	174	8718	97.8%	6.51 [5.57 , 7.62]
Inoue 2019	1	24	0	16	0.2%	2.04 [0.09 , 47.17]
Kitchin 2020	1	59	0	58	0.2%	2.95 [0.12 , 70.96]
**Total (Wald[a])**		**9790**		**9124**	**100.0%**	**6.44 [5.52 , 7.52]**
Total events:	1177		177			

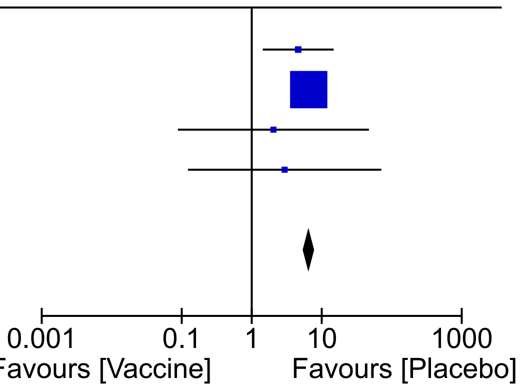

Test for overall effect: Z = 23.56 (P 0.00001)

Heterogeneity: Tau (DL[b]) = 0.00; Chi = 1.08, df = 3 (P = 0.78); I = 0%

Footnotes

[a]CI calculated by Wald-type method.

[b]Tau calculated by DerSimonian and Laird method.

Analysis 5.3: Erythema

Study or Subgroup	Clostridium Difficile Vaccine Events	Clostridium Difficile Vaccine Total	Normal Saline Placebo Events	Normal Saline Placebo Total	Weight	Risk Ratio IV, Random, 95% CI
Christensen 2023	30	985	3	332	1.8%	3.37 [1.04 , 10.97]
Donskey 2024	872	8722	174	8718	97.6%	5.01 [4.27 , 5.88]
Inoue 2019	1	24	1	16	0.3%	0.67 [0.04 , 9.91]
Kitchin 2020	1	59	0	58	0.2%	2.95 [0.12 , 70.96]
**Total (Wald[a])**		**9790**		**9124**	**100.0%**	**4.93 [4.21 , 5.78]**
Total events:	904		178			

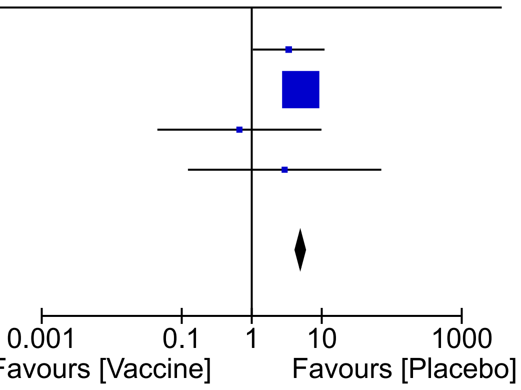

Test for overall effect: Z = 19.79 (P 0.00001)

Heterogeneity: Tau (DL[b]) = 0.00; Chi = 2.65, df = 3 (P = 0.45); I = 0%

Footnotes

[a]CI calculated by Wald-type method.

[b]Tau calculated by DerSimonian and Laird method.

Analysis 5.4: Malaise/Fatigue

Study or Subgroup	Clostridium Difficile Vaccine Events	Clostridium Difficile Vaccine Total	Normal Saline Placebo Events	Normal Saline Placebo Total	Weight	Risk Ratio IV, Random, 95% CI
Christensen 2023	204	985	53	332	34.2%	1.30 [0.99 , 1.71]
Donskey 2024	3227	8722	2877	8718	59.6%	1.12 [1.08 , 1.17]
Inoue 2019	5	24	4	16	4.2%	0.83 [0.26 , 2.64]
Kitchin 2020	12	59	1	58	1.4%	11.80 [1.58 , 87.83]
Sheldon 2016	0	12	1	22	0.6%	0.59 [0.03 , 13.46]
**Total (Wald[a])**		**9802**		**9146**	**100.0%**	**1.20 [0.94 , 1.53]**
Total events:	3448		2936			

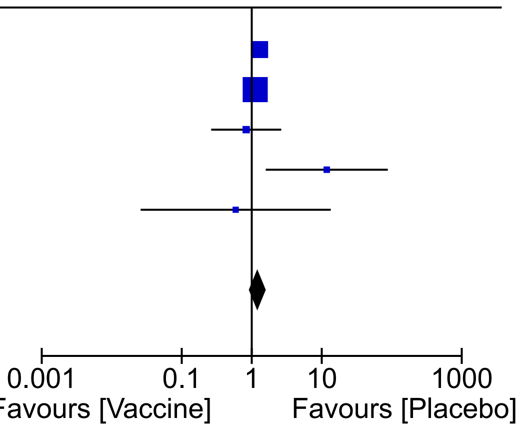

Test for overall effect: Z = 1.46 (P = 0.14)

Heterogeneity: Tau (DL[b]) = 0.03; Chi = 6.74, df = 4 (P = 0.15); I = 41%

Footnotes

[a]CI calculated by Wald-type method.

[b]Tau calculated by DerSimonian and Laird method.
