# Supplementary material for: Comprehensive Evaluation of the Efficacy and Safety of the Clostridioides difficile Toxoid Vaccine: A Meta‐Analysis
Source: Can J Infect Dis Med Microbiol. 2026 Jul 30;2026:1160340. doi: 10.1155/cjid/1160340 (PMC13422635; doi:10.1155/cjid/1160340)
Supplement: Supplementary file 10 — Supporting Information 10 Supporting Figure 9. Forest plots for systemic adverse events (fatigue, myalgia, headache, malaise, arthralgia) in month‐regimen studies receiving 200‐μg vaccine doses. Effect estimates are expressed as RR with 95% CI using a random‐effects model. [file CJID-2026-1160340-s009.pdf]

Analysis 5.5: Myalgia

Study or Subgroup	Clostridium Difficile Vaccine Events	Clostridium Difficile Vaccine Total	Normal Saline Placebo Events	Normal Saline Placebo Total	Weight	Risk Ratio IV, Random, 95% CI
Christensen 2023	74	985	23	332	28.1%	1.08 [0.69 , 1.70]
Donskey 2024	1832	8722	1482	8718	62.7%	1.24 [1.16 , 1.31]
Inoue 2019	3	24	1	16	2.1%	2.00 [0.23 , 17.57]
Kitchin 2020	3	59	10	58	6.0%	0.29 [0.09 , 1.02]
Sheldon 2016	0	12	1	22	1.0%	0.59 [0.03 , 13.46]
**Total (Wald[a])**		**9802**		**9146**	**100.0%**	**1.10 [0.80 , 1.51]**
Total events:	1912		1517			

Test for overall effect: Z = 0.56 (P = 0.58)

Risk Ratio IV, Random, 95% CI

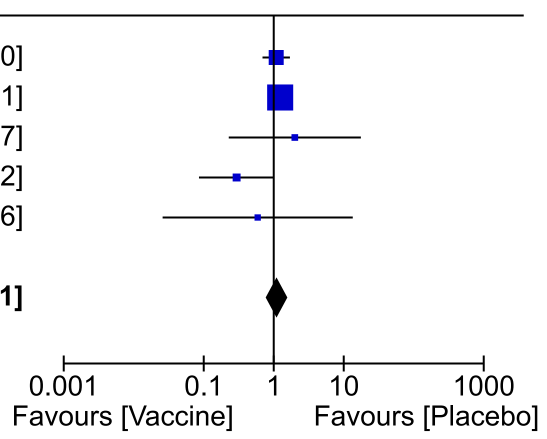

Heterogeneity: Tau (DL[b]) = 0.04; Chi = 5.83, df = 4 (P = 0.21); I = 31%

Footnotes

[a]CI calculated by Wald-type method.

[b]Tau calculated by DerSimonian and Laird method.

Analysis 5.6: Arthralgia

Study or Subgroup	Clostridium Difficile Vaccine Events	Clostridium Difficile Vaccine Total	Normal Saline Placebo Events	Normal Saline Placebo Total	Weight	Risk Ratio IV, Random, 95% CI
Christensen 2023	65	985	15	332	24.0%	1.46 [0.84 , 2.53]
Donskey 2024	1570	8722	1395	8718	70.3%	1.12 [1.05 , 1.20]
Inoue 2019	1	24	4	16	2.4%	0.17 [0.02 , 1.36]
Kitchin 2020	3	59	2	58	3.3%	1.47 [0.26 , 8.50]
**Total (Wald[a])**		**9790**		**9124**	**100.0%**	**1.16 [0.83 , 1.60]**
Total events:	1639		1416			

Test for overall effect: Z = 0.86 (P = 0.39)

Risk Ratio IV, Random, 95% CI

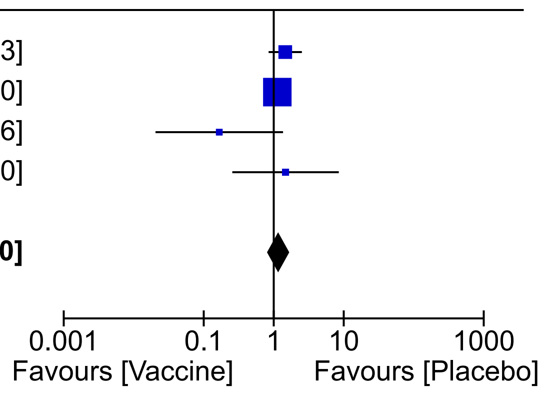

Heterogeneity: Tau (DL[b]) = 0.04; Chi = 4.14, df = 3 (P = 0.25); I = 28%

Footnotes

[a]CI calculated by Wald-type method.

[b]Tau calculated by DerSimonian and Laird method.

Analysis 5.7: Headache

Study or Subgroup	Clostridium Difficile Vaccine Events	Clostridium Difficile Vaccine Total	Normal Saline Placebo Events	Normal Saline Placebo Total	Weight	Risk Ratio IV, Random, 95% CI
Christensen 2023	137	985	32	332	32.8%	1.44 [1.00 , 2.08]
Donskey 2024	2617	8722	2354	8718	66.2%	1.11 [1.06 , 1.16]
Inoue 2019	0	24	0	16		Not estimable
Kitchin 2020	4	59	0	58	1.0%	8.85 [0.49 , 160.77]
**Total (Wald[a])**		**9790**		**9124**	**100.0%**	**1.24 [0.92 , 1.65]**
Total events:	2758		2386			

Test for overall effect: Z = 1.42 (P = 0.15)

Risk Ratio IV, Random, 95% CI

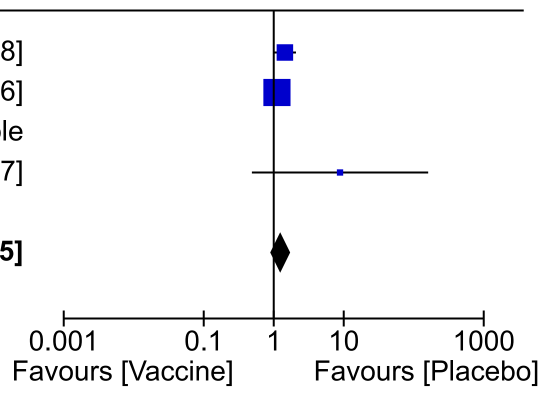

Heterogeneity: Tau (DL[b]) = 0.03; Chi = 3.90, df = 2 (P = 0.14); I = 49%

Footnotes

[a]CI calculated by Wald-type method.

[b]Tau calculated by DerSimonian and Laird method.

Analysis 5.8: Infections and Infestations

Study or Subgroup	Clostridium Difficile Vaccine Events	Clostridium Difficile Vaccine Total	Normal Saline Placebo Events	Normal Saline Placebo Total	Weight	Risk Ratio IV, Random, 95% CI
Christensen 2023	4	983	5	331	55.8%	0.27 [0.07 , 1.00]
Sheldon 2016	2	12	4	22	44.2%	0.92 [0.20 , 4.30]
**Total (Wald[a])**		**995**		**353**	**100.0%**	**0.46 [0.14 , 1.52]**
Total events:	6		9			

Test for overall effect: Z = 1.27 (P = 0.20)

Risk Ratio IV, Random, 95% CI

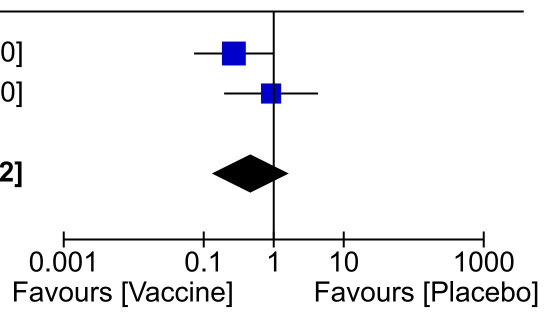

Heterogeneity: Tau (DL[b]) = 0.22; Chi = 1.41, df = 1 (P = 0.24); I = 29%

Footnotes

[a]CI calculated by Wald-type method.

[b]Tau calculated by DerSimonian and Laird method.

Analysis 5.9: Gastrointestinal Disorders

Study or Subgroup	Clostridium Difficile Vaccine Events	Clostridium Difficile Vaccine Total	Normal Saline Placebo Events	Normal Saline Placebo Total	Weight	Risk Ratio IV, Random, 95% CI
Christensen 2023	12	983	3	331	2.5%	1.35 [0.38 , 4.74]
Donskey 2024	174	8722	174	8718	92.9%	1.00 [0.81 , 1.23]
Kitchin 2020	7	59	7	58	4.2%	0.98 [0.37 , 2.63]
Sheldon 2016	0	12	1	22	0.4%	0.59 [0.03 , 13.46]
**Total (Wald[a])**		**9776**		**9129**	**100.0%**	**1.00 [0.82 , 1.23]**
Total events:	193		185			

Test for overall effect: Z = 0.04 (P = 0.97)

Risk Ratio IV, Random, 95% CI

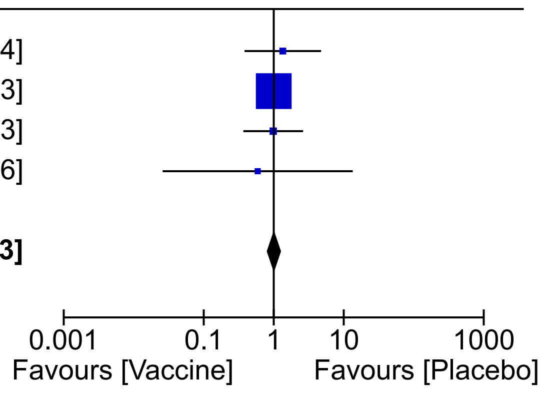

Heterogeneity: Tau (DL[b]) = 0.00; Chi = 0.32, df = 3 (P = 0.96); I = 0%

Footnotes

[a]CI calculated by Wald-type method.

[b]Tau calculated by DerSimonian and Laird method.
