# Supplementary material for: Comprehensive Evaluation of the Efficacy and Safety of the Clostridioides difficile Toxoid Vaccine: A Meta‐Analysis
Source: Can J Infect Dis Med Microbiol. 2026 Jul 30;2026:1160340. doi: 10.1155/cjid/1160340 (PMC13422635; doi:10.1155/cjid/1160340)
Supplement: Supplementary file 11 — Supporting Information 11 Supporting Figure 10. Forest plots for infections, gastrointestinal disorders, musculoskeletal issues, and respiratory disorders in month‐regimen studies receiving 200‐μg vaccine doses. Effect estimates are expressed as RR with 95% CI using a random‐effects model. [file CJID-2026-1160340-s010.pdf]

Analysis 5.10: Skin and Subcutaneous Tissue Disorders

Study or Subgroup	Clostridium Difficile Vaccine Events	Clostridium Difficile Vaccine Total	Normal Saline Placebo Events	Normal Saline Placebo Total	Weight	Risk Ratio IV, Random, 95% CI
Christensen 2023	3	983	4	331	53.3%	0.25 [0.06 , 1.12]
Sheldon 2016	3	12	1	22	46.7%	5.50 [0.64 , 47.27]
**Total (Wald[a])**		**995**		**353**	**100.0%**	**1.06 [0.05 , 21.66]**
Total events:	6		5			

Test for overall effect: Z = 0.04 (P = 0.97)

Heterogeneity: Tau (DL[b]) = 3.85; Chi = 5.32, df = 1 (P = 0.02); I = 81%

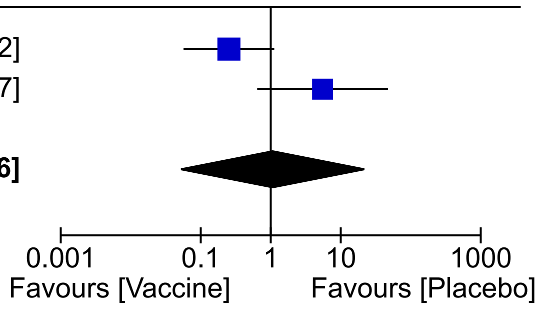

Footnotes
[a]CI calculated by Wald-type method.
[b]Tau calculated by DerSimonian and Laird method.

Analysis 5.11: Respiratory, Thoracic, and Mediastinal Disorders

Study or Subgroup	Clostridium Difficile Vaccine Events	Clostridium Difficile Vaccine Total	Normal Saline Placebo Events	Normal Saline Placebo Total	Weight	Risk Ratio IV, Random, 95% CI
Christensen 2023	79	983	25	331	95.0%	1.06 [0.69 , 1.64]
Sheldon 2016	1	12	5	22	5.0%	0.37 [0.05 , 2.79]
**Total (Wald[a])**		**995**		**353**	**100.0%**	**1.01 [0.64 , 1.59]**
Total events:	80		30			

Test for overall effect: Z = 0.04 (P = 0.97)

Heterogeneity: Tau (DL[b]) = 0.01; Chi = 1.01, df = 1 (P = 0.31); I = 1%

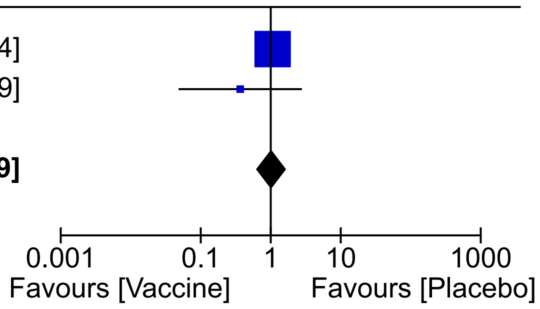

Footnotes
[a]CI calculated by Wald-type method.
[b]Tau calculated by DerSimonian and Laird method.

Analysis 5.12: Bones/Joints/Musculoskeletal System-Related Problems

Study or Subgroup	Clostridium Difficile Vaccine Events	Clostridium Difficile Vaccine Total	Normal Saline Placebo Events	Normal Saline Placebo Total	Weight	Risk Ratio IV, Random, 95% CI
Christensen 2023	16	983	2	331	44.9%	2.69 [0.62 , 11.65]
Sheldon 2016	4	12	3	22	55.1%	2.44 [0.65 , 9.16]
**Total (Wald[a])**		**995**		**353**	**100.0%**	**2.55 [0.96 , 6.81]**
Total events:	20		5			

Test for overall effect: Z = 1.87 (P = 0.06)

Heterogeneity: Tau (DL[b]) = 0.00; Chi = 0.01, df = 1 (P = 0.92); I = 0%

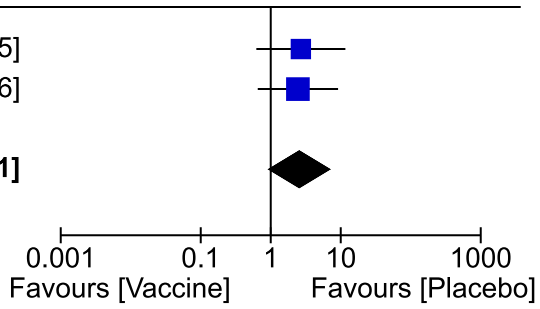

Footnotes
[a]CI calculated by Wald-type method.
[b]Tau calculated by DerSimonian and Laird method.

Analysis 5.13: Serious Adverse Events

Study or Subgroup	Clostridium Difficile Vaccine Events	Clostridium Difficile Vaccine Total	Normal Saline Placebo Events	Normal Saline Placebo Total	Weight	Risk Ratio IV, Random, 95% CI
Christensen 2023	44	983	20	331	1.9%	0.74 [0.44 , 1.24]
Donskey 2024	1297	8722	1267	8718	97.9%	1.02 [0.95 , 1.10]
Inoue 2019	2	24	0	16	0.1%	3.40 [0.17 , 66.48]
Kitchin 2020	3	59	1	58	0.1%	2.95 [0.32 , 27.54]
**Total (Wald[a])**		**9788**		**9123**	**100.0%**	**1.02 [0.95 , 1.09]**
Total events:	1346		1288			

Test for overall effect: Z = 0.51 (P = 0.61)

Heterogeneity: Tau (DL[b]) = 0.00; Chi = 2.99, df = 3 (P = 0.39); I = 0%

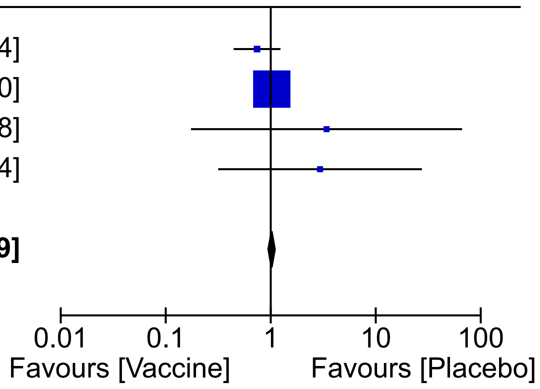

Footnotes
[a]CI calculated by Wald-type method.
[b]Tau calculated by DerSimonian and Laird method.
