# Supplementary material for: Comprehensive Evaluation of the Efficacy and Safety of the Clostridioides difficile Toxoid Vaccine: A Meta‐Analysis
Source: Can J Infect Dis Med Microbiol. 2026 Jul 30;2026:1160340. doi: 10.1155/cjid/1160340 (PMC13422635; doi:10.1155/cjid/1160340)
Supplement: Supplementary file 12 — Supporting Information 12 Supporting Figure 11. Forest plots for overall adverse events, serious adverse events, and mortality in month‐regimen studies receiving 200‐μg vaccine doses. Effect estimates are expressed as RR with 95% CI using a random‐effects model. [file CJID-2026-1160340-s011.pdf]

Analysis 5.14: Mortality

Study or Subgroup	Clostridium Difficile Vaccine Events	Clostridium Difficile Vaccine Total	Normal Saline Placebo Events	Normal Saline Placebo Total	Weight	Risk Ratio IV, Random, 95% CI
Christensen 2023	2	983	2	331	13.8%	0.34 [0.05 , 2.38]
Donskey 2024	212	8722	196	8718	86.2%	1.08 [0.89 , 1.31]
Inoue 2019	0	24	0	16		Not estimable
Kitchin 2020	0	59	0	58		Not estimable
**Total (Wald[a])**		**9788**		**9123**	**100.0%**	**0.92 [0.42 , 2.02]**
Total events:	214		198			

Test for overall effect: Z = 0.21 (P = 0.84)

Heterogeneity: Tau (DL[b]) = 0.18; Chi = 1.35, df = 1 (P = 0.24); I = 26%

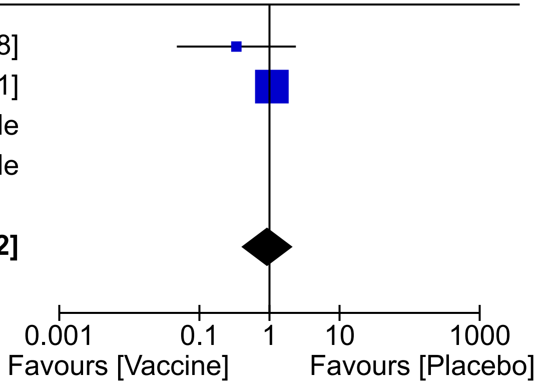

Footnotes

[a]CI calculated by Wald-type method.

[b]Tau calculated by DerSimonian and Laird method.

Analysis 5.15: Adverse Events

Study or Subgroup	Clostridium Difficile Vaccine Events	Clostridium Difficile Vaccine Total	Normal Saline Placebo Events	Normal Saline Placebo Total	Weight	Risk Ratio IV, Random, 95% CI
Christensen 2023	359	983	117	331	2.9%	1.03 [0.87 , 1.22]
Donskey 2024	4546	8722	4443	8718	96.6%	1.02 [0.99 , 1.05]
Inoue 2019	9	24	3	16	0.1%	2.00 [0.64 , 6.27]
Kitchin 2020	16	59	17	58	0.2%	0.93 [0.52 , 1.65]
Sheldon 2016	7	12	13	22	0.2%	0.99 [0.55 , 1.78]
**Total (Wald[a])**		**9800**		**9145**	**100.0%**	**1.02 [0.99 , 1.05]**
Total events:	4937		4593			

Test for overall effect: Z = 1.58 (P = 0.11)

Heterogeneity: Tau (DL[b]) = 0.00; Chi = 1.46, df = 4 (P = 0.83); I = 0%

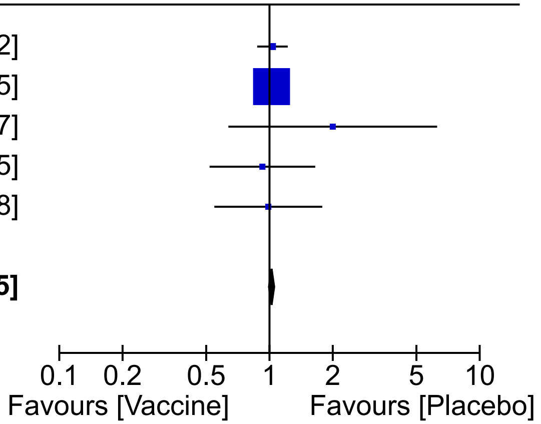

Footnotes

[a]CI calculated by Wald-type method.

[b]Tau calculated by DerSimonian and Laird method.
